# Recurrence of post-traumatic stress disorder: systematic review of definitions, prevalence and predictors

**DOI:** 10.1186/s12888-023-05460-x

**Published:** 2024-01-09

**Authors:** Samantha K Brooks, Neil Greenberg

**Affiliations:** Department of Psychological Medicine, King’s College London, Weston Education Centre, SE5 9RJ London, United Kingdom

**Keywords:** Course, Post-traumatic stress disorder, Predictors, PTSD, Recovery, Recurrence

## Abstract

**Background:**

Many people will experience a potentially traumatic event in their lifetime and a minority will go on to develop post-traumatic stress disorder (PTSD). A wealth of literature explores different trajectories of PTSD, focusing mostly on resilient, chronic, recovered and delayed-onset trajectories. Less is known about other potential trajectories such as recurring episodes of PTSD after initial recovery, and to date there has been no estimate of what percentage of those who initially recover from PTSD later go on to experience a recurrence. This systematic review aimed to synthesise existing literature to identify (i) how ‘recurrence’ of PTSD is defined in the literature; (ii) the prevalence of recurrent episodes of PTSD; and (iii) factors associated with recurrence.

**Methods:**

A literature search of five electronic databases identified primary, quantitative studies relevant to the research aims. Reference lists of studies meeting pre-defined inclusion criteria were also hand-searched. Relevant data were extracted systematically from the included studies and results are reported narratively.

**Results:**

Searches identified 5,398 studies, and 35 were deemed relevant to the aims of the review. Results showed there is little consensus in the terminology or definitions used to refer to recurrence of PTSD. Because recurrence was defined and measured in different ways across the literature, and prevalence rates were reported in numerous different ways, it was not possible to perform meta-analysis to estimate the prevalence of recurrence. We also found no consistent evidence regarding predictors of PTSD recurrence.

**Conclusion:**

A clear and consistent evidence-based definition of recurrence is urgently needed before the prevalence and predictors of recurrence can be truly understood.

**Supplementary Information:**

The online version contains supplementary material available at 10.1186/s12888-023-05460-x.

## Background

Potentially traumatic events are common. Research suggests that over 70% of people will experience a potentially traumatic event (such as witnessing death or serious injury, automobile accident, life-threatening illness or injury, or violent encounter) in their lifetime [1]. Understandably, these events can be very distressing in the short-term and many people will experience acute post-traumatic symptoms in the immediate aftermath of a traumatic event, including intrusive symptoms (e.g. recurrent unwanted thoughts, nightmares); avoidance symptoms (e.g. emotional numbing, social withdrawing); hyperarousal (e.g. easily startled, feeling ‘on edge’); and physical symptoms (e.g. chest pain, dizziness) [2]. For the majority, these symptoms will decline naturally without intervention [3], typically within the first four weeks [2]. An important minority will find their symptoms persist for longer than a month. Those who continue to experience persistent re-experiencing of the traumatic event; avoidance of stimuli associated with the event; negative alterations in cognitions and mood and alterations in arousal and reactivity, causing clinical distress or functional impairment and not attributable to any other medical condition, are likely to be diagnosed with post-traumatic stress disorder (PTSD) [4]. Although only a minority of people who experience potentially traumatic events will go on to develop PTSD, it remains one of the most common mental disorders with lifetime prevalence estimated to be between 8% [5] and 12% [6]. PTSD is associated with reduced health-related quality of life and physical comorbidities, as well as major socio-economic costs [7].

The early 2000s saw a shift from studying PTSD itself as an outcome to studying *change in symptoms* as an outcome [8], with a wealth of studies using modelling approaches such as latent class growth analysis and latent growth mixture modelling to identify different trajectories of PTSD. Most of this literature identifies four trajectories, two of which are relatively stable trajectories (*chronic*, a stable trajectory of post-traumatic stress symptoms, and *resilient*, a stable trajectory of healthy functioning after an adverse event), and two which display dynamic symptom patterns (*recovered*, i.e. decreasing symptoms after an initial diagnosis of PTSD, and *delayed-onset*, i.e. increasing symptoms not meeting the diagnostic criteria for PTSD until potentially months or even years after traumatic exposure) [9]. Van de Schoot et al. [10] suggest that the two trajectories which typically occur less often (chronic and delayed-onset) are at risk of being overlooked by researchers or overwhelmed within the data by the larger trajectories. There may also be other less-researched or less-understood trajectories overlooked to an even greater extent. For example, one previous review [11] identified limited evidence of another, smaller trajectory referred to as a ‘relapsing’ or ‘recurring’ PTSD trajectory, in which individuals develop PTSD, are free from symptoms for long enough to be considered ‘recovered’, and then experience a recurrence of symptoms.

Recurrence is given relatively little attention in the PTSD literature, perhaps due to limitations of study methodologies and the complexities of studying recurrence. For example, Santiago et al. [11] note that few studies of PTSD follow participants for more than a year or with more than two assessments. Clearly, it would not be possible for researchers to identify recurrence of PTSD if data is only collected for two time-points: the only possible outcomes would be low symptom levels at each time-point (‘resilience’), high symptoms at each time-point (‘chronic’), or low level of symptoms at one time-point and a high level at the other (either ‘recovery’ or ‘delayed-onset’ depending on time-point at which symptoms were experienced). Additionally, studies which only follow up participants for a year or less are unlikely to clearly identify a recurrent trajectory of PTSD given the time needed to both recover and to experience a recurrent episode. The timing of PTSD assessment is also important: identification of PTSD recurrence relies on studies capturing the presence of symptoms during the recurrence, rather than before it occurs or after recurring symptoms have subsided. Therefore, it is perhaps unsurprising that the majority of the literature does not identify a ‘recurring’ trajectory of PTSD. Even studies which do identify recurrences often group these in with other trajectories: for example, Mota et al. [12] identified ‘recurrent’ cases of PTSD (individuals who had a lifetime diagnosis in 2002 and another post-2002 diagnosis reported in 2018), but grouped ‘persistent’ and ‘recurrent’ cases of PTSD together. Magruder et al. [13] identified a group of recurrent cases of PTSD – individuals who had lifetime PTSD pre-1992 but not a current diagnosis in 2002, who then had a diagnosis again in 2021, but these were grouped with ‘chronic’ cases. Karamustafalioglu et al. [14] simply include an ‘other’ group constituting both recurrent cases (individuals who met the criteria for PTSD diagnosis 1–3 months post-trauma and at the third follow-up 18–20 months post-trauma, but not at the second follow-up 6–10 months post-trauma) and others with delayed-onset PTSD which resolved. Boe et al. [15] identified a group of individuals with ‘reactivated’ PTSD who reported remission from PTSD in the first five years after the North Sea oil rig disaster of 1980 and a new episode at any point between 1985 and 2007. However, the authors suggest that there are blurred boundaries between delayed-onset and ‘reactivated’ PTSD, going on to include ‘possible delayed cases’ in their analysis of reactivated PTSD.

It is important to note that even the definitions of the more well-established trajectories of PTSD are not without their controversies. For example, Andrews et al. [16] point out the ambiguity in the criterion for delayed-onset PTSD, questioning whether ‘the onset of symptoms’ refers to any symptoms which might eventually lead to PTSD or only to full-blown PTSD itself. North et al. [17] comment on the ambiguities involved in the term *remission* (i.e. whether remission should be symptom-based or threshold-based) as well as the term *onset* (i.e. whether *onset* refers to first symptoms or first meeting diagnostic criteria). Definition of recovery also appears to differ from study to study, with some authors considering recovery to be symptom-based (i.e. no symptoms of the disorder remain) and others considering it to be threshold-based (i.e. some symptoms may remain, but they are beneath the diagnostic threshold) [18].

To date, several systematic reviews have been published which focus solely on only one PTSD trajectory. For example, previous reviews have focused on the delayed-onset trajectory [16, 19]; the recovery trajectory [20]; and the resilient trajectory [21]. To date there has not been a literature review examining evidence of a recurrent trajectory of PTSD. Berge et al. [22] aimed to systematically review research on relapse in veterans but found no studies reporting actual rates of relapse or recurrence. Reviews have also explored the risk of relapse of various anxiety disorders, including PTSD, after discontinuation of antidepressants [23] and after cognitive behavioural therapy [24]. However, there have been no reviews attempting to quantify the risk of PTSD recurring, establish the predictors of recurrence, or quantify how much each predictive factor contributes to the risk of recurrence. The current review aimed to fill this gap in the literature by synthesising existing published data on how researchers define ‘recurrence’ of PTSD, recurrence rates of PTSD, and predictive factors of recurrence.

Having an appropriate understanding of recurrence is important as the concept needs to be properly understood in order to take steps to mitigate the risks of recurrent PTSD episodes. Mitigating the risk of PTSD recurring could benefit the health and wellbeing of trauma-exposed individuals and could reduce the socio-economic costs to the wider society [7]. The prevalence of recurrence is of particular importance to occupational medicine: regularly trauma-exposed organisations, for example, are often faced with decisions about when (and if) staff who have had and recovered from PTSD should return to the frontline duties. Understanding the risk of recurrent episodes may therefore have implications for those in charge of making such decisions. The present time is also a particularly relevant time to develop our understanding of recurrence of PTSD, as it is possible that the COVID-19 pandemic could contribute to recurrence. The pandemic has been declared a potential traumatic stressor, with research suggesting that COVID-19 survivors are at elevated risk of experiencing PTSD [25] and that PTSD symptoms may also develop due to quarantine [26], concerns about the health of loved ones, or economic loss as a result of the pandemic [27]. Hori et al. [28] suggest that the daily television updates regarding COVID-19 could trigger memories of surviving a previous traumatic situation, and exacerbate subthreshold PTSD symptoms. Therefore, experiencing the pandemic could potentially cause a recurrence of symptoms in people who have previously been diagnosed with PTSD.

The aim of this review was to collate literature which provides evidence of the lesser-studied ‘recurrent’ trajectory of PTSD and to identify: (i) the definitions of ‘recurrence’ used throughout the literature; (ii) prevalence of recurrence; and (iii) risk and protective factors for the recurrent trajectory of PTSD.

## Method

This review followed the Preferred Reporting Items for Systematic Reviews and Meta-Analyses (PRISMA) guidelines [29]. Our population of interest were people who had been diagnosed with, recovered from, and experienced a recurrence of PTSD (as diagnosed by a clinician or validated PTSD assessment tool). For the aim relating to prevalence of recurrent episodes, studies needed to involve a suitable design allowing prevalence to be assessed: for example, studies involving a population of people who had recovered from PTSD, followed over time to show how many had a recurrent episode and how many did not. For the other aims (i.e., definitions of recurrence and factors associated with recurrence), a comparison group was not necessary.

### Registering the review

A protocol for the current review was developed and registered with PROSPERO on March 9th 2023 (registration number CRD42023405752). The only deviation from the protocol was the addition of another quality appraisal tool, due to finding a study design (retrospective analysis of existing health data) which we had not anticipated.

### Eligibility criteria

To be included in the review, studies needed to (1) be published in peer-reviewed journals, (2) be published in the English language, (3) use quantitative methodology, (4) use a standardised tool to assess PTSD and (5) present data on recurrence rates of PTSD and/or factors associated with PTSD recurrence. There were no limitations relating to publication date or location of the studies. Case studies were excluded but there were no other exclusion criteria relating to population size.

### Data searching and screening

A systematic literature search was carried out to examine definitions, prevalence rates and predictors of PTSD recurrence. Four electronic databases (Embase, PsycInfo, Medline and Web of Science) were searched on 24th November 2022, using a combination of search terms relating to PTSD, recurrence, and prevalence/predictors which were combined using Boolean operators. The full list of search terms is presented in Appendix 1. The US Department of Veterans Affairs National Center for Post-Traumatic Stress Disorder’s PTSDPubs database (formerly PILOTS) was searched separately on the same date using the individual terms ‘recurrence’ and ‘recurrent’ and limited to peer-reviewed articles. Reference lists of articles deemed to meet the inclusion criteria were also hand-searched.

All citations resulting from the literature searches were downloaded to an EndNote library where duplicates were removed. The titles of all citations were then screened for relevance to the review, with any clearly not relevant being excluded. Abstracts were then screened for eligibility and the full texts of all remaining citations after abstract screening were located and read in their entirety to identify studies meeting all inclusion criteria. The literature searches and screening were carried out by the first author. The two authors met regularly throughout the screening process to discuss any uncertainties about inclusion or exclusion until a decision was reached.

### Data extraction

The first author carried out data extraction of all citations deemed to meet the inclusion criteria. Data were extracted to a Microsoft Excel spreadsheet with the following headings: authors, year of publication, country, study design, sampling method, inclusion/exclusion criteria, study population size, socio-demographic characteristics of participants, type of trauma exposure, time-points at which PTSD was assessed, tools for assessing PTSD, definitions of recovery and recurrence, whether any PTSD treatment was received, prevalence rates of recurrence, and factors examined as potential predictors of recurrence.

### Data synthesis

For the first aim of the review (relating to definitions of recurrence), we designed a table to present data relating to how ‘recurrence’ was understood and defined in each study. The tools used to diagnose and measure PTSD symptoms in the first place are important in understanding how PTSD is defined, so first the assessment tools used in each study were extracted into the table. Given that we wanted to understand the length of time an individual needs to be free of PTSD in order to be considered ‘recovered’, for each study we also included the time-points of PTSD assessment in the table. Next, we included the definitions of recovery and recurrence from each study, explained narratively in the table. We also added information to this table to report whether participants had received PTSD treatment during each study, as some studies focusing on interventions used ‘response to treatment’ in their definitions of recovery. We compared the different definitions used within the studies to establish whether there was consensus within the literature around (i) whether recovery and recurrence are symptom-based or threshold-based and (ii) how long the recovery period between initial diagnosis and recurrent episodes needs to be in order to be considered recurrent rather than chronic PTSD.

The second aim related to prevalence of PTSD recurrence. Due to the various research designs and definitions of ‘recurrence’ in the literature, as well as the different ways in which prevalence was reported, meta-analytic techniques could not be used. Rather, we presented the prevalence data as it was reported in each study. This sometimes meant presenting the prevalence of PTSD recurrence within an entire trauma-exposed population, including those who never experienced PTSD at any time. Other times, this meant presenting the prevalence of PTSD within a population who all had PTSD at one time-point, and other times this meant presenting the prevalence of PTSD within a group who had recovered from PTSD.

Finally, in order to explore factors associated with PTSD recurrence, all variables considered as potential covariates were recorded individually for each study. Each potential predictive factor was descriptively reported in a table, and any found to be significantly associated with experiencing PTSD recurrence were bolded to differentiate between non-significant and significant findings. Factors are also described narratively within the results section. Insights from thematic analysis [30] were used to group similar data together. For example, data relating to gender or age as predictors of recurrence were coded ‘socio-demographic’ and discussed together within the results.

### Quality appraisal

We appraised the quality of studies using National Institutes of Health (NIH) tools: either the Quality Assessment Tool for Observational Cohort and Cross-Sectional Studies or the Quality Assessment of Controlled Intervention Studies tool, depending on study design. Concurrent with other reviews [e.g. 31] we rated quality as ‘poor’ if studies scored 0–4/14, ‘fair’ if they scored 5–10/14 and ‘good’ if they scored 11–14/14. One study used retrospective analysis of existing health data, and for this study we used the MetaQAT Critical Appraisal Tool [32]. To keep the ratings consistent with our rating system for the studies appraised by NIH tools, we defined ‘poor’ quality as a score of 0–34%, ‘fair’ quality as a score of 35–72% and ‘good’ quality as a score of 78% or higher.

## Results

**Fig. 1:**
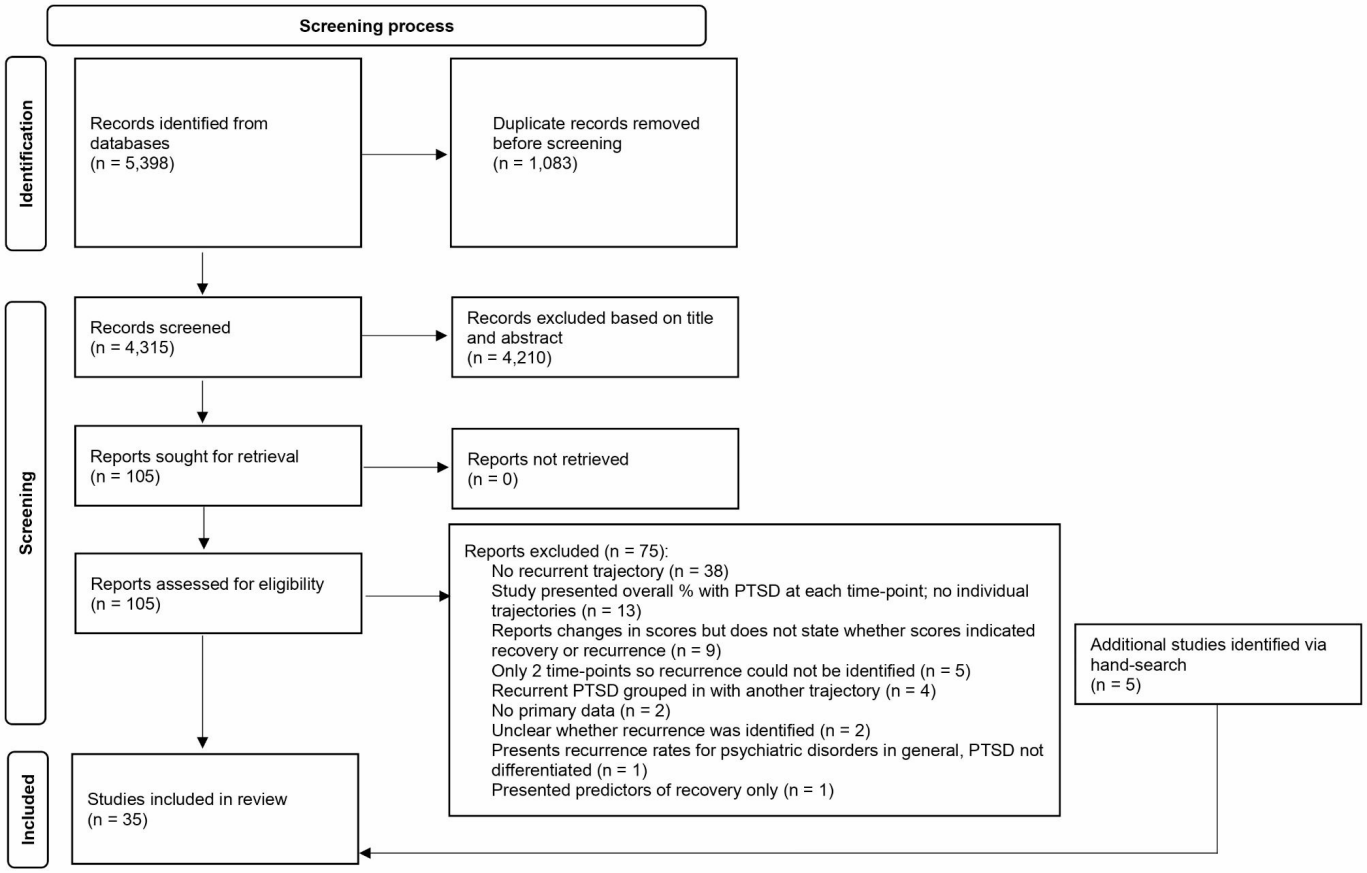
PRISMA flow diagram of screening process

Literature searches yielded 5,398 citations of which 1,083 were duplicates. After title and abstract screening, 4,210 citations were excluded leaving 105 citations for full-text screening. After reading full texts of the remaining citations, 75 were excluded and an additional five studies were added after hand-searching reference lists. A total of 35 citations were included in the review [15, 33–66]. Figure 1 illustrates the screening process in a PRISMA flow diagram.

Table 1 provides an overview of key characteristics of all included studies. Studies originated from the United States of America (n = 13), Denmark (n = 5), Israel (n = 4), China (n = 4), Norway (n = 2), the United Kingdom (n = 2), Japan (n = 1), the Netherlands (n = 1), Switzerland (n = 1), and Turkey (n = 1). The remaining study included participants in multiple different countries across Europe and Asia. Study populations ranged from 35 to 7,918 and included military personnel (n = 15), civilian adults (n = 14), children or adolescents (n = 4) or a combination of military and civilian adults (n = 2). Only three studies were rated as ‘good’ quality; the majority were rated ‘fair’.

**Table 1:** Characteristics of included studies

Authors (year)	Country	Study population	Traumatic exposure	Inclusion / exclusion criteria	Sampling method	Age and gender of participants (at baseline unless otherwise stated)	Quality
An et al. (2022) [33]	China	246 adolescents (T1), 209 (T2), 205 (T3), 121 (T4)	Yancheng tornado in China	None reported	Random sampling of several classes in two schools affected by the tornado	Mean age 14.04, range 12–17 40.7% male, 59.3% female	Fair
Andersen et al. (2014) [34]	Denmark	561 soldiers deployed to Afghanistan in 2009	War in Afghanistan	None reported	Not reported	Mean age: 26.67 low-stable; 22.67 low-fluctuating; 22.20 distress-improving; 25.43 mild distress; 27.56 late onset; 23.18 relieved-worsening Female gender: 4.8% low-stable, 7.1% low-fluctuating, 13.3% distressed-improving, 8.7% mild distress, 0% late onset, 9.1% relieved-worsening	Good
Ansell et al. (2011) [35]	United States of America	499 adults with anxiety disorders from in- and out-patient clinical programs (n = 142 had PTSD diagnosis)	Not reported	Inclusion: Met criteria for anxiety disorder; had at least 12 months of follow-up data available	Participants drawn from a prospective naturalistic longitudinal study; details described in a different publication	Whole sample: mean age 32.5 65% female, 35% male	Fair
Armenta et al. (2019) [36]	United States of America	1,704 US service members and veterans with comorbid PTSD and major depressive disorder	No specific event – military experiences (some may have experienced other trauma unrelated to the military)	Inclusion: Positive screening for both PTSD and major depressive disorder at baseline; completed at least 2 follow-ups	Recruitment process described in a different paper	Mean age 29.1 60.9% male, 39.1% female	Fair
Benítez et al. (2012) [37]	United States of America	199 adults	No specific event – various Traumas most frequently reported were unwanted sex (76.2%), rape (59.8%), being attacked with intent to kill (53.7%), being attacked with a weapon (52.1%), witnessing someone injured or killed (50%), serious accident (47.6%) and fear of being killed/injured (22.3%)	Inclusion: general medical appointment on day of screening; age 18+; English proficiency; meeting DSM-IV criteria for at least one anxiety disorder Exclusion: active psychosis; absence of current address or phone number; pregnancy	Recruitment process described in a different paper	Mean age not reported – all 18+ 80.4% female	Fair
Berntsen et al. (2012) [38]	Denmark	366 soldiers deployed to Afghanistan	N/A – all had been deployed but may have also experienced other traumas	Inclusion: Danish soldiers with a 6-month deployment to Afghanistan Exclusion: None	Entire team of soldiers belonging to the Danish Contingent of the International Security Assistance Force 7 were invited to take part	Mean age 26.59 93.4% male, 6.6% female	Fair
Boe et al. (2010) [15]	Norway	48 disaster survivors	North Sea oil rig disaster	Inclusion: Survivors of the North Sea oil rig disaster, residing in Norway Exclusion: None	All survivors invited to take part	Mean age 59.8 at the 27-year follow-up 100% male	Fair
Chopra et al. (2014) [39]	United States of America	1,185 older adults (n = 81 with PTSD at baseline)	Various – not reported	Inclusion: Age 65+; patients at one of 10 primary care sites across the country; positive screening for depression, anxiety disorders or at-risk drinking Exclusion: Significant cognitive impairment; receiving ongoing mental health treatment	Not reported	Mean age 73.53 544, 304, 102, 66 men PTSD subgroup only: mean age 72.5, 83% male	Fair
Davidson et al. (2005) [40]	United States of America	57 adults with PTSD receiving either fluoxetine (n = 27) or a placebo (n = 30)	Various – trauma included combat (n = 18), sexual trauma (n = 9), other violence (n = 9), bereavement (n = 11), other (n = 10)	Inclusion: met PTSD criteria; discontinued any psychotropic medication for at least 2 weeks before the study; age 18–70 Exclusion: Pregnancy; history of schizophrenia, bipolar disorder, organic brain disease, alcohol or drug abuse/dependence (within past 6 months); ‘mental retardation’; need for ongoing psychotropic medication; significant risk of suicide or suicide attempt in past 6 months; history of significant violence within previous year; medically unstable state; prior nonresponse to adequate treatment with fluoxetine; need for trauma-focused psychotherapy; positive urine drug screen; clinically significant abnormal laboratory tests	Not reported	Mean age 43.5 53.5% female, 46.5% male	Fair
DenVelde et al. (1996) [41]	Netherlands	123 veterans of the Dutch civilian Resistance against the Nazi occupation during World War II	War	Inclusion: Male veterans; receiving a war pension; born between 1/1/1920 and 1/1/1926 No exclusion criteria reported	A sample of individuals receiving a War Pension were randomly selected	Mean age not reported; all participants born between 1/1/1920 and 1/1/1926 100% male	Poor
Fan et al. (2015) [42]	China	T1: 1,573 adolescent (7th or 10th grade) survivors (n = 1,436 at T2; n = 1,288 at T3; n = 1,315 at T4)	Wenchuan earthquake	Inclusion: 7th or 10th graders from schools in Dujiangyan No exclusion criteria reported	All 7th and 10th graders invited to take part	Mean age: 12.3 for the 7th graders, 15.4 for the 10th graders 45.8% male, 54.2% female	Fair
Gonçalves et al. (2011) [43]	United Kingdom	121 women newly diagnosed with ovarian cancer (T1: n = 93; T2: n = 78; T3: n = 69; T4: n = 69; 63 completed assessments at all time points)	Illness: ovarian cancer	Inclusion: new diagnosis of ovarian, peritonea or fallopian tube carcinoma; able to provide informed consent; understanding of English Exclusion: Pre-existing organic brain disorder or severe mental illness	Consecutive new referrals attending a specialist ovarian cancer outpatient clinic were recruited	Mean age 58 100% female	Fair
Gross et al. (2022) [44]	United States of America	2,870 military veterans (admission), 1,848 (discharge), 1,071 (follow-up)	Military	Inclusion: White or Black veterans who initiated PTSD residential treatment during fiscal year 2017; general admission criteria include not currently meeting criteria for an acute psychiatric or medical admission, previous participation in a less restrictive treatment alternative, needing an intensive level of care, not being at significant acute risk of harm to self or others and being capable of basic self-care	All patients invited to take part	Mean age: 42.75 (White participants), 51.26 (Black participants) 11.2% female (White participants), 13.4% female (Black participants)	Fair
Hansen et al. (2017) [45]	Norway	1,974 ministerial employees (T1: n = 1,974; T2: n = 1,780; T3: n = 1,578)	Terrorist attack: bombing of a workplace in Oslo	Inclusion: ministerial employees who were employed in the Norwegian ministries at the time of the attack No exclusion criteria reported	All employees in the ministries at T1 plus former employees working at the time of the attack were invited	Mean age at study start: 45.4 (T1), 45.4 (T2), 45.8 (T3) 42.3% male (T1), 41.2% male (T2), 44% male (T3)	Fair
Hepp et al. (2008) [46]	Switzerland	121 adult hospital patients	Severe injury after accidental injury 59% road traffic accident; 21% sports and leisure-time accident; 14% workplace accident; 6% household accident	Inclusion: age 18–70; sustained accidental injuries that caused life-threatening or critical condition; proficiency in German; clinical condition allowing an extensive clinical interview within 1 month; Injury Severity Score of 10 or more; Glasgow Coma Scale score of 9 or more Exclusion: serious somatic illness; under treatment for mental health immediately prior to accident; marked clinical signs of mental disorders unrelated to accident; attempted suicide; victims of violent assault	Intensive care unit admissions recruited consecutively	Mean age 38.9 77% male, 23% female	Fair
Holliday et al. (2020) [47]	United States of America	7,918 military veterans	Military	Inclusion: veterans who initiated PTSD residential treatment during fiscal years 2015-16; general admission criteria include not currently meeting criteria for an acute psychiatric or medical admission, previous participation in a less restrictive treatment alternative, needing an intensive level of care, not being at significant acute risk of harm to self or others and being capable of basic self-care	All patients	Mean age: 48.79 (those who reported experiencing military sexual trauma), 44.66 (those who did not) Female: 801 + 159 Male: 773 + 6119	Good
Karstoft et al. (2015) [48]	Denmark	561 soldiers (454 who participated in 2.5-year follow-up)	War: Deployment to Afghanistan	Inclusion: Danish soldiers with a 6-month deployment to Afghanistan Exclusion: None	All Danish soldiers who deployed to Afghanistan were asked to participate	Final sample who participated in 2.5-year follow-up: mean age 26.65 94.3% male	Fair
Liang et al. (2019) [49] Liang et al. (2021) [50]	China	301 children at T1 (T2: n = 118; T3: n = 263; T4: n = 253; T5: n = 218)	Wenchuan earthquake	Exclusion: ‘mental retardation’; history of clinically significant head injury; neurological disorders	Convenience sampling	Mean age 12.5 (range 9–14) 52.2% male	Fair
Madsen et al. (2014) [51]	Denmark	456 soldiers who had deployed to Afghanistan	War in Afghanistan	Inclusion: Danish soldiers deployed to Afghanistan February-August 2009	All soldiers deployed to Afghanistan were recruited; this study is a sub-sample with follow-up data on suicidality	Mean age: 26.7 (no suicidal ideation group, n = 394); 26.4 (suicidal ideation group, n = 62) 94.7% male (no suicidal ideation group); 91.9% male (suicidal ideation group)	Fair
Markowitz et al. (2018) [52]	United States of America	43 adults receiving either prolonged exposure (n = 14), relaxation therapy (n = 9) or interpersonal psychotherapy (n = 20) for chronic PTSD	Various – 93% had experienced interpersonal trauma, 62% physical trauma, 35% sexual trauma	Inclusion: Age 18–65; primary DSM-IV diagnosis of chronic PTSD; CAPS score 50+ Exclusion: Psychotic disorders; bipolar disorder; unstable medical conditions; substance dependence; suicidal ideation ; antisocial, schizotypal, borderline or schizoid personality disorder; prior non-response to 8 + weeks of therapy; outside psychotherapy or pharmacotherapy	Patients from a psychiatric institute in New York City recruited via convenience sampling and randomly assigned to an intervention in a 4:4:3 ratio, stratified by presence of major depressive disorder	Mean age 43.21 (prolonged exposure); 37.61 (interpersonal psychotherapy); 42.52 (relaxation) 57% female, 43% male (prolonged exposure); 75% female, 25% male (interpersonal psychotherapy); 78% female, 22% male (relaxation)	Fair
Martenyi et al. (2002) [53]	Belgium, Bosnia (as it was known at the time), Croatia, Yugoslavia (as it was known at the time), Israel, South Africa	131 patients who completed 12 weeks of acute treatment, then re-randomised and continued in a 24-week relapse prevention phase with fluoxetine (n = 69) or placebo (n = 62)	Various – combat-related (44.9% of fluoxetine group, 50% of placebo group), non-combat-related (55.1% of fluoxetine group, 50% of placebo group)	Inclusion: age 18–65; met DSM-IV criteria for PTSD; score of 50 + on the CAPS-DX and score of 4 + on Clinical Global Impression of Severity (CGI-S) scale at baseline Exclusion: Score of 20 + on the Montgomery-Åsberg Depression Rating Scale; serious comorbid illness; concomitant psychotherapy; serious suicidal risk or risk to others; diagnosis of Axis I psychiatric disorder 5 years before traumatic episode; lifetime diagnoses of bipolar disorder, obsessive compulsive disorder or schizophrenia; history of alcohol or substance misuse not resolved at least 6 months prior to the study	Not reported	Mean age 37.1 (fluoxetine), 39.4 (placebo) Fluoxetine group: 78% male, 22% female Placebo group: 84% male, 16% female	Fair
Murphy & Smith (2018) [54]	United Kingdom	960 military veterans	Military	Inclusion: formal diagnosis of PTSD; admitted to a residential intervention; completed at least one full day of paid employment within the UK Armed Forces; if they were taking psychiatric medication at assessment, they had to remain on a stable dose Exclusion: Evidence of significant neurological impairment that would affect ability to engage in therapy; actively psychotic; actively dependent on alcohol or drugs; actively suicidal	All patients	Mean age 42.99 Gender not reported	Fair
Osenbach et al. (2014) [55]	United States of America	194 acutely injured hospitalised civilian trauma survivors	Various – motor vehicle injury (46.91%); assault (20.62%); fall or jump (15.98%); burn (7.22%); sports injury (3.61%); work-related injury (2.58%); other (3.09%)	Inclusion and exclusion criteria reported in a previous publication	Hospitalised trauma survivors randomly assigned to either treatment (intervention focused on early and sustained care of posttraumatic symptoms; n = 96) or usual care (n = 98)	Mean age 39.05 51.03% female	Good
Osofsky et al. (2017) [56]	United States of America	340 patients at rural health clinics	Gulf Oil Spill	Inclusion: receiving integrated services at one of five rural health clinics No other criteria reported	Not reported	Not reported	Fair
Perconte et al. (1991) [57]	United States of America	45 combat veterans with PTSD	Vietnam War	Inclusion: Participation in a partial hospitalisation PTSD treatment programme No other criteria reported	Not reported	Mean age 36.5 Gender not reported	Fair
Sakuma et al. (2020) [58]	Japan	745 local municipality and hospital workers	Great East Japan earthquake	Inclusion: Employed in local municipalities or disaster base hospitals during the earthquake Exclusion: Started employment after the earthquake	All workers invited to take part	Mean age: 43.6 (range 20–66) 58. 9% female, 41.1% male	Good
Solomon & Mikulincer (2006) [59]	Israel	214 veterans from the 1982 Lebanon War (131 with combat stress reaction during the war vs. 83 without)	Lebanon War	Inclusion: Israeli soldiers who had fought in frontline battles in the Lebanon War; combat stress reaction participants had to have been identified as psychiatric casualties by mental health personnel and had a referral for psychiatric intervention during the war and diagnosis of combat stress reaction; comparison group needed to have participated in combat in the same units but not identified as suffering combat stress reaction Exclusion: Indication of other psychiatric disorders	All veterans who met the inclusion criteria were invited to take part	Mean age: 25.81 (range 18–37) Gender not reported	Fair
Solomon et al. (1987) [60]	Israel	35 Israeli soldiers who fought in the 1982 Lebanon War and 1973 Yom Kippur War	Lebanon War and Yom Kippur War	Inclusion: medical files report them as suffering from combat-related PTSD during or immediately after the 1982 Lebanon War; reactivation or exacerbation of symptoms	Case histories of those showing reactivation or exacerbation were hand-selected	Median age 31 (range 28–39) Gender not reported	Fair
Solomon et al. (2018) [61]	Israel	349 veterans and ex-Prisoners of War Ex-Prisoners of War: 164 at T1, 144 at T2, 183 at T3 Veterans: 185 at T1, 143 at T2, 118 at T3	Yom Kippur War	Inclusion: ex-Prisoners of War or veterans of the Yom Kippur War No other criteria reported	All ex-Prisoners of War were approached; a sample of veterans matched to the ex-Prisoners of War in age, rank, military units, psychometric grading and military quality category were drawn from computerised databanks	Mean age 39.8, range 36–50 Gender not reported	Fair
Solomon et al. (2021) [62]	Israel	120 ex-Prisoners of War of the 1973 Yom Kippur War and 136 comparable combat veterans of the same war	Yom Kippur War	Inclusion: Ex-Prisoners of War of the Yom Kippur War or comparable veterans matched on military background and socio-demographic variables who were not taken captive Exclusion: Physical or mental health deteriorated to the point where they could not take part	All ex-Prisoners of War were invited to take part; comparable veterans were sampled from the Israel Defense Forces computerised database	Not reported	Fair
Sørensen et al. (2016) [63]	Denmark	428 Danish soldiers deployed to Afghanistan in 2009 (same cohort as Andersen et al., 2014 and Madsen et al., 2014)	War in Afghanistan	Inclusion: Danish soldiers deployed to Afghanistan from February-August 2009	Whole cohort were recruited; study is a sub-group who completed cognitive assessments and PTSD assessments at follow-up time-points	Mean age 24.0 Gender not reported	Fair
Sungur & Kaya (2001) [64]	Turkey	79 people exposed to the disaster (27 people saved from a burning hotel, 34 people besieged by the fundamentalists, 18 health professionals who treated the injured)	Sivas disaster – a religious fundamentalist protest which caused 37 deaths	Inclusion: Exposure to the disaster No exclusion criteria reported	Not reported	Not reported	Fair
Zanarini et al. (2011) [65]	United States of America	290 adults with borderline personality disorder (vs. 72 with at least one non-borderline axis II disorder)	No specific event – various Of the 163 patients with PTSD at baseline, traumas included childhood sexual abuse (83.4%), adult sexual assault (46%) or both (36.8%) (unclear why these percentages do not add up to 100%)	Inclusion: Patients initially inpatient at a Massachusetts hospital; aged 18–35; known or estimated IQ of 71 or higher; fluent in English Exclusion: History of, or current, symptoms of schizophrenia, schizoaffective disorder, bipolar I disorder or an organic condition that could cause psychiatric symptoms	Not reported	Mean age 27 77.1% female	Fair
Zlotnick et al. (1999) [66]	United States of America	54 adults with anxiety disorders and current PTSD vs. 13 adults with lifetime, but not current, PTSD	No specific event – various Traumatic events included childhood sexual abuse (46%), childhood physical abuse (33%), adulthood rape (22%), adulthood assault (6%), accidental physical harm (7%), witnessing violence in adulthood (4%), witnessing violence in childhood (11%), war (15%), other – including natural disaster, abortion, serious injury to loved one, serious illness and childhood separation from parents (13%)	Inclusion: at least one of panic disorder, agoraphobia, generalised anxiety disorder or social phobia; age 18+; willing and able to consent in participation Exclusion: organic mental disorder; history of schizophrenia; psychosis within last 6 months	Not reported	Current PTSD: Mean age 36 76% female Lifetime PTSD: Mean age 37 62% female	Fair

### Definitions of recurrence

Table 2 reports, for each study, the tools used to assess PTSD; time-points at which PTSD was assessed; definitions of recovery and recurrence; and whether the participants received PTSD treatment or not.

**Table 2:** PTSD assessments and definitions of recovery and recurrence

Study	PTSD assessment	Time-points of assessment	Definition of recovery	Definition of recurrence	Treatment received?
An et al. (2022) [33]	17-item Child PTSD Symptom Scale (CPSS)	6, 9, 12 and 18 months after the tornado	PTSD symptoms decreasing from above the cut-off at T1 to T2 to below the cut-off at T3	PTSD symptoms increasing from below the cut-off at T3 to above the cut-off at T4 (trajectory labelled ‘recurrent dysfunction’)	Not reported
Andersen et al. (2014) [34]	PTSD Checklist, Civilian Version (PCL-C); Structured Clinical Interview for DSM-IV (T6 only)	T1: 5–6 weeks pre-deployment T2: During deployment T3: 1–3 weeks post-deployment T4: 2 months post-deployment T5: 7 months post-deployment T6: 2.5 years post-deployment	Temporary ‘symptom relief’ during deployment	‘Drastic symptom increase’ following the temporary relief and continuing through 2.5 years (labelled ‘relieved-worsening’ trajectory)	Some participants reported to have psychological or psychiatric treatment after deployment; data not presented
Ansell et al. (2011) [35]	Structured Clinical Interview for DSM-IV Axis I Disorders – Patient Version; weekly ‘psychiatric status ratings’	Weekly ratings for 7 years	Remission defined as eight consecutive weeks with minimal or no symptoms	Relapse defined as, after remission, experiencing four consecutive weeks with symptoms meeting full diagnostic criteria	All participants were recruited from in- and outpatient clinical programmes
Armenta et al. (2019) [36]	PTSD Checklist-Civilian Version (PCL-C)	Baseline (T1) and four follow-up assessments, approximately every 3 years between 2001–2016	Lower level of symptoms at first follow-up	Symptoms steadily worsened over the remainder of the study period (labelled ‘relapse’ trajectory)	Not reported
Benítez et al. (2012) [37]	Structured Clinical Interview for DSM-IV, patient version	T1 (intake) and follow-up interviews at 1-year intervals for 5 years	8 consecutive weeks of PTSD at a Psychiatric Status Rating (PSR) level of 2 or less	Onset of PTSD symptoms at a PSR level of 5 or greater for 2 consecutive weeks following a recovery period	Medication – SSRI (25.1%); Medication – benzodiazepines (12.1%); Psychosocial treatment (33.2%)
Berntsen et al. (2012) [38]	Posttraumatic Stress Disorder Checklist (PCL)	Symptoms assessed pre-deployment; during the 6-month deployment; 1–3 weeks after return; 2–4 months after return; and 7–8 months after return	No explicit definition of recovery – latent class growth modelling revealed trajectories which showed ‘temporary beneficial effects associated with deployment’, i.e. ‘high or moderate levels of PTSD symptoms at pre-deployment [which] decreased either at deployment, return from deployment, or approximately 3 months after return’	No explicit definition of recurrence – latent class growth modelling revealed trajectories where temporary reductions in symptoms associated with deployment later ‘increased again after 3 or 7 months following return’	Not reported
Boe et al. (2010) [15]	Structural Clinical Interview for DSM-IV axis I Disorders (SCID-I)	Symptoms measured 5.5 months, 14 months, 5 years and 27 years after the disaster	Not reported	Either fulfilling all DSM-IV diagnostic criteria or sub-syndromal cases of PTSD, referred to as ‘reactivated PTSD’	Not reported
Chopra et al. (2014) [39]	PTSD assessment schedule based on DSM-IV criteria of PTSD	Symptoms assessed at baseline, 3 months and 6 months	Participants ‘denied any reexperiencing symptoms’	Meeting DSM-IV criteria for PTSD	Participants were attending primary care clinics – unclear what treatment was provided
Davidson et al. (2005) [40]	Clinical Global Impressions of Severity (CGI-S); Short PTSD Rating Interview (SPRINT); Davidson Trauma Scale	Baseline, 1-week, 2-week, 4-week and then monthly follow-ups over 1 year	CGI scores of 1 (very much improved), 2 (much improved) or 3 (moderately improved)	CGI score reverted back to no improvement relative to baseline or worse (i.e. scores of 4 or greater), or which had increased by at least 2 points relative to improvement status at Week 24; a participant was also considered relapsed if an ‘untoward clinical event’ occurred such as psychiatric hospitalisation, suicidality, or the patient dropping out because they did not feel progress had been sustained	Randomised trial – 30 participants received a placebo and 27 received fluoxetine; medication and support were provided as the ‘only form of intervention’
DenVelde et al. (1996) [41]	28-item Dutch self-rating scale based on the DSM-III criteria for PTSD; participants rated PTSD symptoms as ‘none’, ‘mild’, ‘moderate’, ‘severe’ or ‘very severe’	Participants gave complete life-history data at one timepoint (exact date not provided; sometime between 1985–1994)	Participants self-reported whether their PTSD symptoms were chronic/progressive, lasted for less than 5 years, or entailed ‘remissions and exacerbations’	See previous column	Not reported
Fan et al. (2015) [42]	Posttraumatic Stress Disorder Self-Rating Scale	6, 12, 18 and 24 months post-earthquake	‘Relapsing/remitting’ group characterised by PTSD symptoms fluctuating and showing a cyclical course across the follow-up period	See previous column	Not reported
Gonçalves et al. (2011) [43]	Posttraumatic Stress Diagnostic Scale (PDS)	T1: beginning of cancer treatment; T2: 6–8 weeks later; T3: 15 weeks after T1; T4: 27 weeks after T1	Not meeting the full criteria for PTSD case diagnosis	Meeting criteria for PTSD diagnosis (one reexperiencing symptom, three avoidance symptoms, two arousal symptoms, duration of symptoms for at least 1 month and impairment in at least one area of functioning)	Of those with PTSD diagnoses, 5% reported current psychological help, 16% reported past psychological help and 30% reported use of antidepressants
Gross et al. (2022) [44]	PCL-5	Admission to residential treatment, discharge (mean length of stay 51 days, range 3-322), and follow-up (4 months post-discharge)	Significant decrease in PTSD symptoms over the course of treatment	Not defined	All participants were admitted to residential rehabilitation treatment programmes although the length of time they stayed varied
Hansen et al. (2017) [45]	Norwegian version of the Posttraumatic Stress Disorder Checklist-Specific (PCL-S)	10, 22 and 34 months post-bombing	Not meeting the criteria for symptom-defined PTSD at T2	Meeting the criteria for PTSD at T3	Not reported
Hepp et al. (2008) [46]	Impact of Events Scale; Clinician Administered PTSD Scale for DSM (CAPS)	2 weeks, 6 months, 12 months and 36 months post-accident	N/A	‘Secondary increase of the CAPS score above 30 later on’	6 patients at T4 (7%) reported psychopharmacological and/or psychotherapeutic treatment related to the accident
Holliday et al. (2020) [47]	PCL-5	Admission to treatment, discharge (length of treatment not reported), 4-month follow-up	Clinically meaningful change in PTSD symptoms from admission to discharge	Not defined	All participants were admitted to residential rehabilitation treatment programmes although only around three-quarters completed the programme
Karstoft et al. (2015) [48]	PTSD Checklist, civilian version (PCL-C)	Pre-deployment, during deployment, on return from deployment, + 3 months, + 7 months, + 2.5 years	Moderate initial symptom level that *‘decreased somewhat during deployment’*	Moderate initial symptom level that ‘decreased somewhat during deployment’ and then *increased drastically after deployment*	Some participants had received psychological or psychiatric help before deployment
Liang et al. (2019) [49] Liang et al. (2021) [50]	20-item section of the UCLA PTSD reaction index for DSM-IV (Steinberg et al., 2004)	4, 16, 29, 40 and 52 months post-earthquake	Relapsing trajectory: High PTSD score at T1 which *decreased to a low level* until T3 but then increased again at T4	Relapsing trajectory: High PTSD score at T1 which decreased to a low level until T3 but then *increased again* at T4	Not reported, although one of the two schools is described as having ‘better psychological intervention and peer social support networks’
Madsen et al. (2014) [51]	PCL-C	6 weeks pre-deployment; during deployment; at homecoming; and 3 months, 7 months and 2.5 years post-deployment	‘Relieved-worsening’ trajectory is not clearly defined	See previous column	Not reported
Markowitz et al. (2018) [52]	CAPS; Structured Clinical Interview for DSM-IV; “other measures”	T1: start of treatment T2: 14 weeks later T3: 26 weeks from baseline	Response to treatment defined as: ≥30% decrement from baseline CAPS score and remission as CAPS score < 20	Relapse defined as: <30% reduction from baseline CAPS score after having met response criteria at week 14	10 weekly 90-minute sessions, over 14 weeks, of prolonged exposure helping the patient to reconstruct a narrative of the trauma, understand the rationale for facing reminders of the trauma, constructing a hierarchy of fears, and undergoing imaginal and in vivo exposure *Or* 14 weekly 50-minute sessions of individual psychotherapy focusing on the interpersonal consequences of trauma and using affect as a guide to handling interpersonal encounters *Or* 9 weekly 90-minute sessions of relaxation therapy where therapists induced muscle and mental relaxation
Martenyi et al. (2002) [53]	Pre-treatment: Structured Clinical Interview for DSM-IV Axis I Disorders for Patients, Investigator Version (SCID-I modified); Clinician-Administered PTSD Scale, Current Diagnostic Version (CAPS-DX) Follow-ups: TOP-8 scale; CGI-S scale; CAPS-DX; Clinical Global Impression of Improvement (CGI-I); Davidson Trauma Scale	12 weeks after treatment, 36 weeks after treatment (unclear how long after trauma exposure)	‘Responding to treatment’ defined as: 50% decrease in the eight-item Treatment Outcome PTSD (TOP-8) score from baseline; CGI-S score of 2 or less; and failing to meet DSM-IV diagnostic criteria for PTSD	Relapse criteria: 40% increase in TOP-8 score, increase in CGI-S score of 2 + from week 12; or the ‘clinical judgement of the investigator’	12 weeks double-blind treatment with fluoxetine or placebo followed by 24-week relapse prevention (re-randomised to either fluoxetine or placebo) for those who responded to treatment
Murphy & Smith (2018) [54]	Impact of Events Scale-Revised (IES-R)	Intake, end of six-week treatment, and then 6 weeks, 6 months, and 12 months after the end of treatment	Latent class growth analysis identified a ‘response-remit’ trajectory, with significant reductions in PTSD observed post-treatment	Latent class growth analysis identified a ‘response-remit’ trajectory, with PTSD severity scores returning to pre-treatment levels ‘at the first follow-up point 6 weeks later and at subsequent timepoints’	All participants had been admitted to a residential intervention
Osenbach et al. (2014) [55]	PCL-C	Baseline measures completed in hospital; follow-up assessment 1–3 weeks after injury; additional follow-up measures at 1, 3, 6, 9 and 12 months post-injury	The relapsing/remitting trajectory showed ‘moderate symptoms that varied slightly across time, but stayed relatively moderate’	See previous column	96/194 received a series of interventions focused on early, sustained care of posttraumatic symptoms, beginning with motivational interviewing and behavioural activation and then ‘stepped up’ to higher-intensity care (pharmacotherapy)
Osofsky et al. (2017) [56]	PCL-C	Intake and at 1, 3, 6, 12 and 18 month follow-up intervals	‘Initial declines’ – not reported whether this meant eradication of symptoms or not meeting the threshold	‘Increasing symptoms’ – again, not reported what this means; not clear whether those in the increasing group did have clinically significant PTSD scores	Participants were receiving ‘integrated services’ at health clinics but it is not clear what this means
Perconte et al. (1991) [57]	Clinician diagnosis according to DSM-III criteria; Minnesota Multiphasic Personality Inventory (MMPI), including the PTSD subscale	Pre-treatment, termination of treatment, and 18-month follow-up	Obtaining at least a 1-point improvement on the BHPRS Global Pathology Index	Being hospitalised at least once since termination of treatment	Partial hospitalisation programme which involved group psychotherapy, flooding/desensitisation techniques, problem-solving and stress management, social skills and assertiveness training, substance abuse counselling, individual counselling, vocational counselling, recreational activities Veterans attended treatment 3 days per week for 4–6 h
Sakuma et al. (2020) [58]	Japanese version of the PTSD Checklist Specific Version (PCL-S)	14, 30, 43 and 54 months post-earthquake	‘Fluctuating course’ defined as: cyclical course moving above and *below the diagnostic threshold*	‘Fluctuating course’ defined as: cyclical course moving *above* and below *the diagnostic threshold*	Not reported
Solomon & Mikulincer (2006) [59]	PTSD Inventory based on the DSM-III	1, 2, 3 and 20 years post-war	Not meeting criteria for PTSD	No formal definition was provided. However, a recurrent trajectory could be seen as some participants met the criteria for PTSD at one time-point, then did not meet the criteria, then met the criteria again at a later time-point	All participants in the combat stress reaction group had undergone at least some treatment
Solomon et al. (1987) [60]	Clinician’s diagnosis, confirmed by the authors using DSM-III criteria	Time-points unclear – analysis of ‘case histories’ between 1973 and 1982	‘Completely dormant or resolved episode of combat stress’	Reactivation of a dormant/resolved episode or exacerbation of residual symptoms of combat stress reaction	Not reported, but it is clear from the case studies provided as examples that some had been ‘referred for psychiatric treatment’
Solomon et al. (2018) [61]	PTSD-Inventory, a 17-item self-report scale corresponding to DSM PTSD criteria	T1: 1991 T2: 2003 T3: 2008 T4: 2014	Observed trajectories included “recovery followed by delayed-onset PTSD” and “recovery followed by delayed-onset PTSD followed by recovery”; unclear what this means or what the criteria for meeting these trajectories were	See previous column	Not reported
Solomon et al. (2021) [62]	PTSD-Inventory, a 17-item self-report scale corresponding to DSM PTSD criteria	18, 30, 35, 42 and 47 years post-war	Participants who ‘endorsed PTSD criteria’ in earlier waves but not later waves	‘Reactivated PTSD’ group: participants who initially had PTSD, recovered, then had a ‘reactivation’ of PTSD at a later measurement; the parameters of reactivation are not defined	Not reported
Sørensen et al. (2016) [63]	PCL-C	6 weeks pre-deployment; during deployment; at homecoming; and 3 months, 7 months and 2.5 years after deployment	‘Relieved-worsening’ trajectory had initial decreasing PTSD symptoms followed by a steep increase in symptoms post-deployment	See previous column	Not reported
Sungur & Kaya (2001) [64]	Diagnosis based on the presence of each symptom of PTSD criteria for DSM-III-R	1 month, 6 months, 12 months and 18 months post-disaster	‘Acute recurrent’ group defined as: symptomatic at T1, *no symptoms at T2*, symptomatic again at T3 and/or T4	‘Acute recurrent’ group defined as: symptomatic at T1, no symptoms at T2, *symptomatic again at T3 and/or T4*	Some participants took antidepressants but most did not seek treatment
Zanarini et al. (2011) [65]	Structured Clinical Interview for DSM-III-R Axis I disorders	Baseline, 2-year follow-up, 4-year follow-up, 6-year follow-up, 8-year follow-up	‘Remission’ defined as any 2-year period in which the criteria for PTSD were no longer met	Any 1-month period in which the criteria for PTSD were met after a 2-year remission	All participants were in inpatient treatment at the start of the study; around 90% were in individual therapy and taking psychotropic medications at baseline and around 70% were in therapy or taking medications at follow-ups
Zlotnick et al. (1999) [66]	Baseline: Structured Clinical Interview for the DSM-III-R Patient Version (SCID-P; Spitzer et al., 1988) Follow-ups: Longitudinal Interval Follow-up Evaluation to collect data on the course of illness. Participants were assigned weekly Psychiatric Status Ratings corresponding to the number and frequency of PTSD symptoms experienced during the week	6-month intervals for the first 2 years and yearly thereafter, until 5 years after intake	‘Remission’ defined as ‘minimal or no symptoms of PTSD for at least 8 consecutive weeks’	Participants described as having a ‘re-occurrence of PTSD at follow-up’ but no definition is provided	Participants described as ‘in treatment at intake’ but type of treatment is not specified and the authors report that they did not obtain information on whether the treatment was specifically for PTSD

### Terminology

The first aim of the review was to explore how ‘recurrence’ is defined in the literature. We found no consensus in terms of how this is defined. In fact, the studies used a variety of different terms to describe the emergence of new PTSD episodes after initial ‘recovery’, including ‘recurrence’ [33, 37, 44, 47, 64, 65]; ‘relapse’ [35, 36, 40, 49, 50, 52, 53, 57]; ‘reactivation’ [15, 60, 62]; ‘exacerbation/reactivation’ [61]; ‘relieved-worsening PTSD’ [34, 48, 51, 63]; ‘response-remit’ trajectory [54]; ‘fluctuating course’ [58]; ‘intermittent cases’ [43]; ‘delayed increase in symptoms’ [46]; and the ‘relapsing/remitting’ trajectory [42, 55]. Many others simply described recurrence as ‘symptom increase’ [38], ‘initial declines followed by symptom increases’ [56] or ‘exacerbation of symptoms’ [41, 60]. Some studies did not name the trajectory at all; rather, they presented tables or flow charts showing the number of participants with PTSD at each time-point, from which it was possible for us to identify a sub-group of participants who were described as having PTSD at one time-point, not having it at least one follow-up, and then having it again at subsequent time-points [39, 59]. Similarly, Hansen et al. [45] identified and commented on a sub-group of participants who met the criteria for PTSD, did not meet the criteria at a subsequent time-point, and then met the criteria again later, but they did not give this a name.

### Criteria for recurrence

Several studies defined recurrence (or equivalent terminology such as relapse) as meeting diagnostic criteria for PTSD at a follow-up time-point after an initial ‘recovery’ period where they did not meet the cut-off for PTSD [33, 35, 37, 39, 43, 45, 46, 58, 65]. Holliday et al. [47] referred to ‘clinically meaningful change in PTSD symptoms’, which was also assumed to refer to clinical cut-off scores. Markowitz et al. [52] based the definition of relapse on similarity to baseline scores. Sungur and Kaya [64] defined recovery and recurrence as being asymptomatic and then symptomatic again, but it is not clear whether this referred to clinical cut-offs. One study defined ‘reactivation’ of PTSD as meeting full diagnostic criteria *or* being a sub-syndromal case [15]. Others were more vague and did not mention cut-offs, instead referring to dramatic or steep symptom increases [34, 38, 56, 63], fluctuating symptoms [42, 55], returning to pre-treatment levels of PTSD [54], symptoms which ‘decreased somewhat and increased drastically’ [48], symptoms which ‘decreased to a low level and increased again’ [49, 50] or ‘steadily worsening’ symptoms [36]. DenVelde et al. [41] simply asked participants to self-report whether they had ‘experienced remissions and exacerbations’. Martenyi et al. [53] had multiple definitions of relapse, including increases in scores on their PTSD measures or ‘the clinical judgement of the investigator’. Others labelled the trajectory but did not specify the parameters of their definitions [51, 60–62, 66]. One study [57] used ‘being hospitalised’ as a proxy measure of PTSD recurrence, although this way of defining recurrence would obviously not capture individuals who developed recurring symptoms which were not severe enough to warrant hospitalisation; additionally, no criteria for hospitalisation were described. Similarly, Davidson et al. [40] described ‘relapse’ as PTSD scores reverting back to baseline or worse, or experiencing an ‘untoward clinical event’ including suicidality, hospitalisation, or dropping out of the study due to feeling progress was not being made.

We found little consensus as to how long participants needed to be symptom-free (or have reduced symptoms) in order to be considered ‘recovered’ prior to recurrence. The majority of studies simply based their definitions on the time-points of the study, suggesting that recurrence was identified if participants had PTSD at baseline, did not have PTSD during at least one follow-up, and then had PTSD again at a later follow-up. The time-points of follow-ups ranged from weeks to months to years. Only four studies suggested specific timeframes: three studies claimed that participants needed to be ‘recovered’ for eight weeks in order for later reports of PTSD to count as ‘recurrence’ rather than symptom fluctuation [35, 37, 66] whereas Zanarini et al. [65] reported that participants needed to be not meeting the PTSD criteria for at least two years in order to be considered ‘recovered’. Similarly, most studies did not clarify a time-scale for how long symptoms needed to be experienced in order to be considered a ‘recurrence’. Most studies again simply based their diagnosis on the scores participants happened to report on the days they were assessed. Few studies specified a time-frame: three [35, 43, 65] suggested a duration of four consecutive weeks of meeting their criteria for PTSD, while Benítez et al. [37] suggested two weeks of symptoms was sufficient to identify a recurrent episode.

### Prevalence of recurrence

The review’s second aim was to explore PTSD recurrence rates. Table 3 presents data on the prevalence of recurrence of PTSD for each study. The second column of Table 3 presents the data that is reported in the original studies. The findings reported in this column are not easily comparable because studies reported recurrence rates in different ways. Some reported the *percentage of the entire trauma-exposed sample* who experienced PTSD recurrence (column 3 of Table 3). Others reported *the percentage of those with PTSD who experienced recurrence* (column 4 of Table 3) and the remaining studies reported *the percentage of those who recovered from PTSD who experienced recurrence* (column 5 of Table 3). Three studies [44, 47, 57] did not report the prevalence of recurrence, but were still included in the review as they included definitions and/or predictors of recurrence. One study [60] deliberately chose a sample who had all experienced recurrence; therefore, recurrence prevalence data for this study was not recorded in Table 3 as it would, by design, be 100%.

**Table 3:** Prevalence of recurrence

Study (population)	Data relating to prevalence of recurrent PTSD, as presented in the studies	Prevalence of recurrence in entire trauma-exposed sample (including those who never developed PTSD)	Prevalence of recurrence in people with PTSD	Prevalence of recurrence in people who recovered from PTSD
An et al. (2022) (adolescents) [33]	‘Recurrent dysfunction’ trajectory: 91 participants **(37%)** vs. recovery trajectory (n = 107, 43.5%) and delayed dysfunction (n = 48, 19.5%)	37%	-	-
Andersen et al. (2014) (military) [34]	Relieved-worsening: **2.0%** (n = 11) vs. low-stable 78.1%; low-fluctuating 7.5%; mild distress 4.1%; distressed-improving 2.7%; late onset 5.7%	2%	-	
Ansell et al. (2011) (civilian) [35]	Of the 142 participants with PTSD at baseline, 76.8% (n = 102) remitted by Year 7 and of these **34%** had relapsed by Year 7	-	-	34%
Armenta et al. (2019) (military) [36]	‘Relapse’ group: **24.5%** vs. rapid recovery (31.1%), chronic (26.1%), and gradual recovery (18.3%)	-	24.5%	-
Benítez et al. (2012) (civilian) [37]	Of the 199 participants with PTSD, 38% (n = 62) had a remission by year 5. Of these 62, **29.5%** had a recurrence episode by year 5	-	-	29.5%
Berntsen et al. (2012) (military) [38]	‘Late-benefit group’ (PTSD pre-deployment, which increased slightly during deployment and on return, before dropping to subthreshold levels 3 months after return and then increasing again 7 months after return): n = 14, **4%** ‘Strong-benefit group’ (high level of PTSD pre-deployment which decreased during deployment, dropped to subthreshold levels on return, and then increased at both 3 months and 7 months after return): n = 8, **2%** vs. resilient (low levels at all times) (n = 306, 84%); new-onset (n = 14, 4%); ‘mild-benefit group’ (PTSD pre-deployment which decreased to subthreshold levels on return and then slightly worsened between 3–7 months post-return; n = 24, 7%). The mild-benefit group has not been included as an example of recurrence as while their symptoms worsened again after initial decline, they did not appear to have reached threshold levels)	6%	-	-
Boe et al. (2010) (civilian) [15]	‘Reactivated PTSD’ was observed in **18.8%** (n = 9) survivors, of which 4.2% (n = 2) were possible delayed-onset cases. In 5 of the reactivated cases (**10.4%)** the PTSD episode fulfilled all DSM-IV diagnostic criteria whilst 4 (8.3%) were sub-syndromal cases vs. 58.3% (n = 28) resilient, 14.6% (n = 7) remitted, 8.3% (n = 4) chronic Of the 9 cases of reactivated PTSD, 3 had experienced additional traumatic events (meeting the stressor criterion in DSM-IV). Precipitating events (not necessarily meeting the stressor criterion) included accident (n = 2), loss (n = 3), reminder (i.e. event that resembled or was directly associated with the original trauma, n = 2), physical illness (n = 1), or none identified (n = 1) All of the reactivated cases had additional lifetime psychopathologies: recurrent depression (n = 5), alcohol abuse or addiction (n = 6), phobia (n = 3), panic disorder (n = 2), single depressive episode (n = 3)	18.8%	-	-
Chopra et al. (2014) (civilian) [39]	Of the 81 participants with baseline PTSD, 9 reported ‘trauma only’ (no reexperiencing symptoms) at 3 months and **2/9** (22.2%) had PTSD again at 6 months. Of the 81 with PTSD at baseline, 4 reported ‘no trauma’ at 3 months and **2/4** (50%) met PTSD criteria again at 6 months. (Some confusion arises over why participants who had experienced PTSD in the past would then claim they had experienced ‘no trauma’. The authors attribute this to fluctuations in recall of previously experienced traumatic events.) Therefore overall, of the 81 participants with baseline PTSD, 4 (4.9%) experienced recurrence.	-	4.9%	-
Davidson et al. (2005) (military and civilian) [40]	Relapses in the placebo group: **50%** (n = 15) Relapses in the fluoxetine group: **22.2%** (n = 6) Estimated relative risk for relapse was 1.55 (placebo) and 0.44 (fluoxetine). Odds ratio was 3.50 for relapse on placebo relative to fluoxetine	-	-	50% (placebo group) 22.2% (fluoxetine group)
DenVelde et al. (1996) (military) [41]	**13.8%** (n = 17) with PTSD onset between 1944–1950 and **35.8%** (n = 44) with PTSD onset after 1950 experienced ‘remissions and exacerbations’. That is, 61/123 (**49.6%)** of those with PTSD experienced recurrences. Other trajectories included chronic progressive (9.6%, n = 12 onset 1944–1950, 12.2%, n = 15 post-1950 onset) and duration of symptoms less than 5 years (8.1%, n = 10 onset 1944–1950, 20.3%, n = 25 post-1950 onset)	**-**	49.6%	**-**
Fan et al. (2015) (adolescents) [42]	‘Relapsing/remitting’ group (PTSD symptoms fluctuating and showing a cyclical course across the follow-up period): **3.3%** vs. resistance (65.3%), chronic dysfunction (7.2%), recovery (20%), delayed dysfunction (4.2%)	3.3%	-	-
Gonçalves et al. (2011) (civilian) [43]	PTSD trajectories were examined for 63 participants who completed PTSD measures at all time-points. Intermittent cases (met PTSD criteria at least one time point but not all) accounted for **57%** (n = 36); of these, 4 met criteria at T1 and T3, 2 met criteria at T2 and T4, 3 met criteria at T1, T2 and T4, and 4 met criteria at T1, T3 and T4 vs. stable non-cases (never meeting criteria) (30%, n = 19) and persistent cases (PTSD at all timepoints) (13%, n = 8).	57%	-	-
Gross et al. (2022) (military) [44]	Recurrence rate not reported	-	-	-
Hansen et al. (2017) (civilian) [45]	Of the 123 employees present during the bomb explosion, **3.3%** (n = 4) experienced recovery and recurrence (i.e. PTSD at T1, no PTSD at T2, PTSD at T3) vs. 65.9% no PTSD at any time; 0.8% PTSD at T3 only; 1.6% PTSD at T2 only; 1.6% at T2 and T3; 8.9% PTSD at T1 only; 4.9% PTSD at T1 and T2; 13% PTSD at all time-points Of the 814 employees not present during the explosion, **0.2%** (n = 2) experienced recovery and recurrence (i.e. PTSD atT1, no PTSD at T2, PTSD at T3) vs. 93.7% no PTSD at any time; 0.7% PTSD at T3 only; 1.4% PTSD at T2 only; 0.3% at T2 and T3; 2.3% PTSD at T1 only; 0.7% PTSD at T1 and T2; 0.5% PTSD at all time-points	3.3% (those present during explosion) 0.2% (those not present during explosion)	-	-
Hepp et al. (2008) (civilian) [46]	Of those with a CAPS score of 30 or more at any time (n = 25, 28%): ‘Delayed increase’ group: n = **7 (28%)** vs. initial increase in symptoms (n = 10), initial decrease in symptoms (n = 8)	-	28%	-
Holliday et al. (2020) (military) [47]	Recurrence rate not reported	-	-	-
Karstoft et al. (2015) (military) [48]	‘Relieved-worsening trajectory’ (moderate initial symptom level that decreased somewhat during deployment and then increased drastically after deployment): **2.0%** vs. low-stable (78.1%), low-fluctuating (mild symptoms before and after deployment, 7.5%), mild distress (low symptoms before deployment followed by moderate increase after deployment, 4.1%), late onset (5.7%), distress-improving (moderate symptoms pre-deployment which decreased after deployment, 2.7%)	2%	-	-
Liang et al. (2019) [49] Liang et al. (2021) [50] (adolescents)	‘Relapsing’ trajectory (high PTSD score at T1 which decreased to a low level until T3 but then increased again at T4): **17.7%** vs. resilient trajectory (74.9%) and recovery trajectory (7.5%)	17.7%	-	-
Madsen et al. (2014) (military) [51]	‘Relieved-worsening’ trajectory: n = **9/456 (1.9%)** vs. low-stable (n = 359); late onset (n = 29); low-fluctuating (n = 30); distressed-improving (n = 12); mild distress (n = 17)	1.9%	-	-
Markowitz et al. (2018) (civilian) [52]	Of the 43 participants who responded to treatment and completed follow-up measures, 23 were remitters and 20 were responders; 3 months later, 27 had achieved remission status, 10 had responder status and **6 (14% of the original 43)** had relapsed – including 2 responders to interpersonal psychotherapy, 1 responder to prolonged exposure, 1 remitter to relaxation and 1 responder to relaxation	-	-	14%
Martenyi et al. (2002) (military and civilian) [53]	Fluoxetine/fluoxetine group: **5.8%** (4/69) experienced clinical relapse (57 completed without relapse, 1 discontinued due to adverse event, 3 chose to leave study, 4 were non-compliant) Fluoxetine/placebo group: **16.1%** (10/62) experienced clinical relapse (41 completed without relapse, 3 were lost to follow-up, 2 chose to leave study, 6 were non-compliant)	-	-	5.8% (fluoxetine group) 16.1% (placebo group)
Murphy & Smith (2018) (military) [54]	Of 960 participants, **1.2%** were in the response-remit class vs. 2.7% displayed low start-high response; 27.5% with a resistant trajectory; 22.9% showed high start-high response; 47.5% showed high start-moderate response	1.2%	-	-
Osenbach et al. (2014) (civilian) [55]	Relapsing/remitting trajectory: **35%** vs. resilience (28%); chronic (27%); recovery (10%)	35%	-	-
Osofsky et al. (2017) (civilian) [56]	‘Initial declines followed by increasing symptoms’: n = 108, **32%** vs. steep decreasing symptoms (n = 79, 23%); stable high symptoms (n = 56, 17%); stable low symptoms (n = 97, 29%)	32%	-	-
Perconte et al. (1991) (military) [57]	Does not report how many participants are in the relapsed group	-	-	-
Sakuma et al. (2020) (civilian) [58]	‘Fluctuating course’ (cyclical course moving above and below the diagnostic threshold): **3.5%** (n = 26) vs. resistance (never showing more than mild distress; 62.7%); sub-syndromal (elevated symptoms below diagnostic threshold; 24.3%); recovery (initially above threshold symptoms but decreasing to normal level over time; 6.3%); and chronic (pronounced symptoms from the onset; 3.2%)	3.5%	-	-
Solomon & Mikulincer (2006) (military) [59]	Of the 131 participants with a combat stress reaction, a recurrent trajectory was observed in **32/131** (24.4%) participants: PTSD at T2 and T4 (n = 1); PTSD at T1, T2 and T4 (n = 17); PTSD at T1 and T3 (n = 2); PTSD at T1 and T4 (n = 7); PTSD at T1, T3 and T4 (n = 5) Of the 83 with no combat stress reaction, a recurrent trajectory was observed in **11/83 (13.2%)** participants: PTSD at T2 and T4 (n = 6); PTSD at T1, T2 and T4 (n = 2); PTSD at T1 and T3 (n = 1); PTSD at T1 and T4 (n = 2); PTSD at T1, T3 and T4 (n = 0)	24.4% of those with a combat stress reaction 13.2% of those without a combat stress reaction	-	-
Solomon et al. (1987) (military) [60]	(Note that this was a study specifically of individuals with reactivated PTSD, so there is no comparison group who did not have a recurrence) ‘Uncomplicated reactivation’ (individuals diagnosed as having a combat stress reaction which they had completely recovered from and were symptom-free and then developed full-blown PTSD after being exposed to a battle situation in a subsequent war): **23%** (n = 8) ‘Heightened vulnerability: specific sensitivity’ (individuals who succeeded in professional and social functioning despite persistent minor symptoms, for whom stimuli reminiscent of the original trauma retained the power to reactivate symptoms): **51%** (n = 18) ‘Heightened vulnerability: moderate generalised sensitivity’ (individuals for whom symptoms of PTSD remained apparent in civilian settings, but continued to serve in the reserves despite this, who experienced intense anticipatory anxiety when orders for the 1982 war were issued): **9%** (n = 3) ‘Heightened vulnerability: severe generalised sensitivity’ (individuals who displayed inability to function in any setting and whose conditions worsened upon call-up to the 1982 war): **17%** (n = 6)	-	-	-
Solomon et al. (2018) (military) [61]	“Recovered followed by delayed-onset PTSD”: 11 **(3.5%);** “recovered followed by delayed-onset followed by recovered”: 1 **(0.3%)** (unclear what this means) vs. resilient (52.1%); chronic (1.9%); delayed-onset at T2 (11.3%); delayed-onset at T3 (8.7%); delayed-onset at T4 (3.2%); recovered at T2 (2.2%); recovered at T3 (0.3%); recovered at T4 (0.6%); delayed-onset followed by recovery (14.9%) (It should be noted that a three-class solution represented the best fit for the data; chronic vs. resilient vs. delayed-onset)	3.8%	-	-
Solomon et al. (2021) (military) [62]	Between Waves 1–4: ‘Reactivation’ group (participants who initially had PTSD, recovered, then had a reactivation of PTSD at a later measurement): **1.6%** (n = 3) vs. chronic (met PTSD criteria at all waves, 5.4%); delayed (did not endorse PTSD criteria in first wave but did in later wave/s, 35.1%); recovered (endorsed PTSD criteria in the first wave but not in later wave/s, 3.2%); and resilient (never met the criteria for PTSD, 54.6%) At Wave 5: **16.7%** of those who had initially recovered had PTSD again during the COVID-19 pandemic	1.6	-	16.7%
Sørensen et al. (2016) (military) [63]	Of 384 soldiers with data on trajectories: Relieved-worsening trajectory: n = 9 (**2.1%** of the sample) vs. low stable (77.3%); low fluctuating (7.6%); mild distress (3.9%); distress improving (3.1%); late onset (5.5%)	2.1%	-	-
Sungur & Kaya (2001) (civilian) [64]	‘Acute recurrent’ group (symptomatic at T1, no symptoms at T2, symptomatic again at T3 and/or T4): **8.9%** (n = 7) vs. no disorder (50.5%); acute PTSD (symptomatic at T1 only; 11.4%); chronic resolved (symptomatic at T1 and T2 only, 1.3%); chronic persistent (symptomatic at all time points, 11.4%); delayed persistent (symptomatic at T2, T3 and T4, 5%); symptoms at 6-months only (1.3%); symptoms at 12 months only (1.3%); symptoms at 18 months only (8.9%)	8.9%	-	-
Zanarini et al. (2011) (civilian) [65]	Recurrence (defined as meeting the PTSD criteria for one month after a two-year period of remission) occurred in **40%** (n = 30) of participants with borderline personality disorder and PTSD at baseline who had experienced a remission (25% by the 4-year follow-up, 36% after both six and eight years, and 40% by the 10-year follow-up). Recurrence also occurred in 6/15 axis II comparison participants who met criteria for PTSD at baseline and had a remission during the follow-up years.	-	-	40%
Zlotnick et al. (1999) (civilian) [66]	Of 13 participants with lifetime PTSD, **2/13 (15.4%)** had a recurrence of PTSD during the follow-up; neither remained in their episode of PTSD during the subsequent follow-up interval	-	15.4%	-

Most studies (19/35) reported the prevalence of recurrence within the entire trauma-exposed population. We would therefore expect prevalence rates to be extremely small, given that the majority of trauma-exposed people will not develop PTSD in the first place [3], let alone have recurrent episodes. However, in several studies this was not the case. Prevalence of recurrence ranged from 0.2% (for a sub-set of participants who did not directly witness the disaster in question) [45] to 57% of 63 women newly-diagnosed with ovarian cancer [43]. The latter study was carried out over 27 weeks and identified ‘intermittent cases’ who had PTSD at one time-point, no PTSD at a later time-point, and then PTSD again later on. We note that 27 weeks is a fairly short period of time for both recovery and recurrence to occur, and it is therefore possible that the data reflects symptom fluctuations rather than true recovery or recurrence. Overall, the mean prevalence of recurrent PTSD in trauma-exposed populations was 13.1%, and the median was 3.8%.

Five studies presented the prevalence of recurrence within populations diagnosed with PTSD. We would expect these prevalence rates to be higher than the prevalence rates of recurrence within full trauma-exposed samples, as they are based on populations who developed PTSD only. The rates were 4.9% [39], 15.4% [66], 24.5% [36], 28% [46] and 49.6% [41]. Mean and median prevalence of recurrent PTSD were both 24.5%.

Seven studies presented data on the prevalence of recurrence within sub-sets of study populations who had *recovered from* PTSD; therefore, the only possible trajectories for these participants would be recurrence or maintenance of recovery. Recurrence rates ranged from 5.8% (for a sub-set of participants treated with fluoxetine) [53] to 50% (for a sub-group treated with a placebo) [40]. Mean prevalence of recurrent PTSD was 25.4% and the median was 22.2%.

The three studies rated highest in quality [34, 47, 55] did not report similar findings relating to prevalence. Holliday et al. [47] did not present prevalence data at all. Andersen et al. [34] reported that 2% of participants followed the ‘relieved-worsening’ trajectory, whereas Osenbach et al. [55] reported that 35% of participants followed the ‘relapsing-remitting’ trajectory. Notably, Andersen et al.’s [34] participants were military personnel, whilst Osenbach et al.’s [55] participants were civilian trauma survivors. For this reason, we decided to look separately at recurrence rates in military and civilian participants. We also decided to look separately at data on children as children’s experiences during and after potentially traumatic events are likely to be distinct from those of adults [67]. Table 4 presents the mean and median recurrence rates for different populations.

**Table 4:** Mean and median recurrence rates by population

Population	Mean recurrence rate (entire trauma-exposed population)	Median recurrence rate (entire trauma-exposed population)	Mean recurrence rate (population with PTSD)	Median recurrence rate (population with PTSD)	Mean recurrence rate (population who recovered from PTSD)	Median recurrence rate (population who recovered from PTSD)
All studies	13.1% (n = 21)	3.8% (n = 21)	24.5% (n = 5)	24.5% (n = 5)	25.4% (n = 9)	22.2% (n = 9)
Military populations only	5.82% (n = 10)	2.1% (n = 10)	37.1% (n = 2)	37.1% (n = 2)	16.7% (n = 1)	16.7% (n = 1)
Civilian adult populations only	19.8% (n = 8)	13.9% (n = 8)	16.1% (n = 3)	15.4% (n = 3)	29.4% (n = 4)	31.8% (n = 4)
Military and civilian populations	-	-	-	-	23.5% (n = 4)	19.2% (n = 4)
Children and adolescents	19.3% (n = 3)	17.7% (n = 3)	-	-	-	-

### Prevalence of PTSD recurrence in military populations

Fifteen studies focused on military personnel and veterans, three of which did not provide prevalence data and one of which included *only* participants with PTSD recurrence. Military studies which presented rates of recurrence in trauma-exposed populations (rather than focusing on people diagnosed with PTSD only) typically found low prevalence of recurrence: seven studies found prevalence rates under 4% [34, 48, 51, 54, 61, 62]. Another study found a prevalence rate of 6% [38]. The only higher prevalence rates were reported by Solomon & Mikulincer [59], who reported recurrence rates of 24.4% for those with combat stress reactions (people referred for psychiatric intervention during the war) and 13.2% for participants who participated in combat in the same units but without need for psychiatric intervention during the war. This study assessed participants over twenty years, which may explain its higher prevalence rate than the majority of studies which were completed within two-and-a-half years or less. However, the study period was shorter than the forty-seven years of Solomon et al.’s [62] study, which reported only a 1.6% rate of recurrence. It is unclear why Solomon and Mikulincer [59] found much higher rates of recurrence.

Two military studies reported recurrence rates for PTSD-populations. These were 24.5% [36] and 49.6% [41]. We note that all of Armenta et al.’s [36] participants had comorbid depression at baseline. We also note some concerns about the reliability of DenVelde et al.’s study [41], which was a retrospective study asking participants to give complete life-history data at one time-point only.

One military study reported on the prevalence of recurrence in a sub-group of participants who had recovered. Solomon et al. [62], who reported a prevalence rate of 1.6% (out of the entire trauma-exposed sample) over the first forty-two years of the study, found in a follow-up at forty-seven years that 16.7% of those who had initially recovered experienced recurrence of PTSD during the COVID-19 pandemic.

### Prevalence of PTSD recurrence in civilian adult populations

Fourteen studies focused on civilian adults. Findings relating to recurrence prevalence in entire trauma-exposed samples were mixed. Two studies reported rates of under 5% [45, 58] in survivors of a terrorist attack and an earthquake respectively. Sungur and Kaya [64] reported a recurrence rate of 8.9% in survivors of the Sivas disaster, a religious fundamentalist protest which resulted in civilian deaths. Higher rates of recurrence were reported for survivors of an oil rig disaster (18.8%) [15], survivors of an oil spill (32%) [56], acutely injured trauma survivors (35%) [55] and women recently diagnosed with ovarian cancer (57%) [43].

For populations of civilians with PTSD only, recurrence rates were 4.9% [39] (type of trauma not reported), 15.4% [66] (trauma type varied), and 28% [46] (participants severely injured in accidents). Four studies reported data on the prevalence of recurrence in populations who had previously recovered from PTSD. Reported rates were 14% [52] (trauma type varied), 29.5% [37] (trauma type varied), 34% [35] (trauma type not reported) and 40% [65] (trauma type varied).

### Prevalence of PTSD recurrence in children

Four studies focused on recurrence in adolescents / children, with mixed findings. Fan et al. [42] found that 3.3% of 1,573 earthquake survivors experienced ‘relapsing/remitting’ PTSD. Liang et al. [49, 50] found that 17.7% of 301 earthquake survivors experienced the ‘relapsing’ trajectory of PTSD. An et al. [33] found that 37% of 246 adolescents experienced ‘recurrent dysfunction’ after experiencing an earthquake.

### Prevalence of PTSD recurrence in combined military and civilian populations

Finally, two studies included both military and civilian participants; both of these studies were trials comparing fluoxetine to placebo treatment in people with PTSD. Davidson et al. [40] found that half of the placebo group relapsed after recovery, compared to 22.2% of the fluoxetine group. Martenyi et al. [53] reported lower rates of ‘relapse’: 16.1% of the placebo group and 5.8% of the fluoxetine group. The latter study followed up participants after 36 months, while Davidson et al. [40] followed up participants for a year after treatment.

### Predictors of PTSD recurrence

The third and final aim of the present review was to identify factors associated with PTSD recurrence. Firstly, we note that (as shown in Table 2), participants in a number of studies had received some type of intervention during the study period, which was typically not accounted for in analyses of predictors. Many other studies did not report whether participants received treatment or not. Having treatment, whether it be medication, therapy, or a combination, is likely to be an important factor influencing PTSD trajectory, given that there are evidence-based treatments for the condition [68], but this was typically not explored.

Table 5 shows the factors considered as predictors in each study, with significant associations presented in bold. The majority of included studies (22/35) explored at least one covariate; the remaining studies either did not explore covariates or combined recurrent trajectories with other trajectories in their analyses of predictors. Of those studies which did explore covariates of recurrence, we found little consensus.

**Table 5:** Predictive factors

Study	Factors associated with recurrence (significant relationships in bold)
An et al. (2022) [33]	Compared with the recovery trajectory: **Female gender, Grade 9 in school (vs. 7 or 8), academic burnout**, subjective fear, property loss, post-traumatic growth Compared with the delayed trajectory: **Female gender, Grade 9 in school (vs. 7 or 8)**, academic burnout, subjective fear, property loss, **lower post-traumatic growth**
Andersen et al. (2014) [34]	N/A – all ‘fluctuating’ trajectories were combined into one for analysis of predictors
Ansell et al. (2011) [35]	Age; gender; Axis I disorder; major depressive disorder; schizotypal personality disorder; avoidant personality disorder; **(no baseline** **diagnosis of) obsessive-compulsive personality disorder**; borderline personality disorder
Armenta et al. (2019) [36]	Compared with rapid recovery group: Age, gender, race, marital status, education, service branch, service component, pay grade, **combat deployment, childhood physical abuse**, childhood sexual abuse, childhood verbal abuse, childhood neglect, sexual assault, physical assault, disabling injury/illness, other life events, **obesity**, smoking status, alcohol problems, sleep duration, social support, other anxiety syndrome, bodily pain, somatic symptoms
Benítez et al. (2012) [37]	N/A – only predictors of recovery are explored
Berntsen et al. (2012) [38]	N/A – the three groups who experienced ‘benefits’ of deployment in terms of reduction of PTSD symptoms were grouped together in regression analysis
Boe et al. (2010) [15]	Number of intrusion symptoms 5.5 months after the disaster, number of avoidance symptoms 5.5 months after the disaster, **number of intrusion symptoms 14 months after the disaster, number of avoidance symptoms 14 months after the disaster, number of intrusion symptoms 5 years after the disaster, number of avoidance symptoms 5 years after the disaster** When each residual symptom was considered separately, effect sizes were found for **sleeping difficulties due to images or thoughts, bad dreams about the event, staying away from reminders, trying not think about the event (at 14 months)** and **waves of strong feelings, things that made them think of the event, thinking about the event when they didn’t mean to, images popping into their mind, sleeping difficulties due to images or thoughts, being aware of feelings but not dealing with them (at 5 years)**
Chopra et al. (2014) [39]	‘Recurrent’ groups were too small to feature in analysis of associated factors
Davidson et al. (2005) [40]	**Treatment group (placebo vs. fluoxetine)**
DenVelde et al. (1996) [41]	N/A
Fan et al. (2015) [42]	Compared to the recovery group, relapsing participants experienced **fewer negative life events at 6-months post-earthquake but more such events at 24 months, received less social support at 24 months** No significant differences in gender, school grade, number of children in family, having a family member injured/killed/missing, house damage, property loss, directly witnessing the disaster, social support at 6 months, positive coping at 6 months, negative coping at 6 months
Gonçalves et al. (2011) [43]	N/A – intermittent and persistent cases combined
Gross et al. (2022) [44]	Although race was significantly associated with treatment response (with Black veterans experiencing an attenuated response over the course of the PTSD programme) and Black veterans had significantly greater PTSD symptoms at discharge, there were no significant differences between Black and White participants in terms of symptom recurrence
Hansen et al. (2017) [45]	N/A
Hepp et al. (2008) [46]	N/A
Holliday et al. (2020) [47]	Veterans who experienced military sexual trauma had ‘modestly greater recurrence of symptoms’ but this does not appear to be significant
Karstoft et al. (2015) [48]	**Poor adjustment to civilian life (i.e. difficulties with community reintegration after deployment)** was significantly higher for the relieved-worsening group than for all other groups
Liang et al. (2019) [49] Liang et al. (2021) [50]	**Greater trauma severity; School 2 rather than School 1;** gender; school grade; pre-quake trauma
Madsen et al. (2014) [51]	**Suicidal ideation** was significantly higher in the relieved-worsening group than the low-stable group; the relieved-worsening group had the highest rate of suicidal ideation (66.7%)
Markowitz et al. (2018) [52]	N/A
Martenyi et al. (2002) [53]	**Discontinuation of fluoxetine** (fluoxetine group were significantly less likely to relapse than placebo group, especially for those with **combat-related PTSD**)
Murphy & Smith (2018) [54]	Response-remit group vs. resistant group: baseline depression; baseline anxiety; magnitude of reexperiencing symptoms; magnitude of avoidance symptoms; magnitude of hyperarousal symptoms; combat exposure
Osenbach et al. (2014) [55]	**Recurrent life stressors** increased the odds of membership in chronic, relapsing-remitting or recovery group trajectories vs. resilient; Intervention (vs. **usual care**) associated with membership in relapsing-remitting or chronic trajectory vs. resilient; Relapsing-remitting not associated with race; psychiatric history; depressive symptoms
Osofsky et al. (2017) [56]	Compared to the stable-low group: **higher stress relating to the oil spill; higher number of traumas; physical abuse; emotional abuse; domestic violence; meeting the cut-off for PTSD**
Perconte et al. (1991) [57]	Vs. improved group: Age; combat exposure; months spent in Vietnam; previous hospitalisations; weeks enrolled in treatment; pre-treatment Psychiatric Scale ratings; family support; **higher weekly alcohol intake both before and at termination of treatment;** number of treatment sessions attended; **higher somaticism, obsessive-compulsive symptoms, depression, anxiety, hostility, phobic anxiety and psychoticism**
Sakuma et al. (2020) [58]	Doing mainly disaster-related work; **lack of communication;** lack of rest; displacement; dead or missing family members; near-death experience; pre-disaster treatment for physical illness; **pre-disaster treatment for mental illness**
Solomon & Mikulincer (2006) [59]	**Combat stress reaction** – odds of combat stress reaction casualties to relapse are significantly higher than those of veterans without antecedent combat stress reaction
Solomon et al. (1987) [60]	N/A
Solomon et al. (2018) [61]	N/A
Solomon et al. (2021) [62]	N/A
Sørensen et al. (2016) [63]	Vs. the low stable group, the relieved-worsening group had significantly **lower cognitive ability scores**
Sungur & Kaya (2001) [64]	N/A
Zanarini et al. (2011) [65]	Baseline predictors of time-to-recurrence among patients with borderline personality disorder: presence of history of childhood sexual abuse, **severity of childhood sexual abuse, adult rape history at baseline, combination of a history of childhood sexual abuse and adult rape history, sexual assault during 10 years of prospective follow-up**
Zlotnick et al. (1999) [66]	N/A

### Sociodemographic factors

Gender was considered as a potential covariate by six studies; one [33] found that recurrent PTSD was associated with female gender while five studies (including two based on the same data-set) [49, 50] found no significant gender association [35, 36, 42, 49, 50]. None of the three studies testing age as a covariate found a significant association [35, 36, 57]. One study of school-aged children found that children in a higher grade (i.e. older in age) were more likely to experience PTSD recurrence [33], while three studies of two cohorts [42, 49, 50] found no significant association between recurrence and school grade. Three studies considered race as a covariate, finding no significant association between PTSD recurrence and race [36, 44, 55]. Other socio-demographic characteristics considered included number of children in the family [42], marital status and level of education [36], none of which were found to be associated with PTSD recurrence. For military participants, there were no significant differences in service branch, service component or pay grade between the recurrent and rapid recovery groups [36].

### Psychiatric history

Seven studies considered psychiatric history and concurrent diagnoses as potential covariates of PTSD recurrence, again with mixed findings. Recurrence was not found to be associated with other anxiety syndromes [36], baseline levels of anxiety [54], depressive symptoms [55], baseline levels of depression [54] or psychiatric history [55]. Ansell et al. [35] found that diagnoses of a number of co-morbid mental health disorders such as major depressive disorder and personality disorders such as schizotypal personality disorder, avoidant personality disorder and borderline personality disorder were not associated with recurrence, but participants with a baseline diagnosis of obsessive-compulsive personality disorder were significantly less likely to experience PTSD recurrence. Conversely, Perconte et al. [57] found that those who experienced recurrence were significantly more likely to report obsessive-compulsive symptoms than those whose symptoms improved without recurrence. Sakuma et al. [58] found that pre-disaster treatment for mental illness was significantly associated with PTSD recurrence, but note that the results should be interpreted carefully due to the very small number of participants in the ‘fluctuating symptoms’ group who appeared to have experienced recurrent episodes. Perconte et al. [57] found that, versus the improved symptoms group, those with PTSD recurrence were more likely to report depression, anxiety, hostility, phobic anxiety, somaticism and psychoticism; however, previous psychiatric hospitalisations and pre-treatment ratings of global pathology on a psychiatric scale did not predict recurrence. Finally, Madsen et al. [51] found that suicidal ideation was significantly higher in the ‘relieved-worsening PTSD’ group than the ‘low-stable’ group and that suicidal ideation was in fact highest in the recurrent (termed ‘relieved-worsening’) group than any other. However, it should be noted that suicidality was not assessed at baseline in this study, therefore it is not clear whether suicidal ideation is a cause or a consequence of PTSD recurrence.

### Physical health

Fewer studies considered physical health as a potential predictor of PTSD recurrence. One study found no association between recurrence and disabling injury/illness, somatic symptoms or bodily pain [36] and another found no association between recurrence and prior treatment for physical illness [57]. However, obesity was a significant predictor of PTSD recurrence [36]. In terms of health-related behaviours, Armenta et al. [36] found no association between PTSD recurrence and smoking status, alcohol problems or sleep duration. However, Perconte et al. [57] found that higher weekly alcohol intake both before and at termination of PTSD treatment predicted recurrence.

### Cognitive ability

Only one study [63] explored cognitive ability as a potential covariate, finding that the participants who were in the recurrent (termed ‘relieved-worsening PTSD’) group had significantly lower cognitive ability scores than those in the ‘low-stable’ group.

### Trauma history and pre-trauma experiences

The review also found mixed evidence for trauma history as a predictor of PTSD recurrence. Liang et al. [49, 50] found no association between pre-disaster traumatic experience and PTSD recurrence. Armenta et al. [36] found no association between recurrence and childhood sexual abuse, childhood verbal abuse, childhood neglect, sexual assault, physical assault, or ‘other life events’, but did find that participants reporting a history of childhood physical abuse were significantly more likely to experience PTSD recurrence. Holliday et al. [47] found that veterans who had experienced military sexual trauma (MST) had greater initial reductions in PTSD symptoms than those who had not experienced MST, but also experienced a ‘modestly greater’ recurrence of symptoms than those without MST, although this difference did not appear to reach statistical significance. Zanarini et al. [65] found that the presence of childhood sexual abuse history did not significantly predict time-to-recurrence, but severity of childhood sexual abuse, adult rape history, combination of childhood sexual abuse history and adult rape history, and experiencing sexual assault during study follow-up were associated with less time-to-recurrence. Osofsky et al. [56] found that abuse, emotional abuse, domestic violence, and greater number of traumas experienced were associated with recurrence of PTSD, and Osenbach et al. [55] found that recurrent life stressors significantly increased the odds of membership in chronic, relapsing or recovery groups rather than the resilient group. For military participants, one study found combat deployment was significantly associated with recurrent PTSD [36] while others found combat exposure was not associated with recurrence [54, 57]. Finally, Fan et al. [42] found that compared to the recovery group, relapsing participants experienced significantly fewer negative life events 6-months post-disaster, but significantly more such events at the 24-month follow-up.

Few other pre-trauma experiences were considered. An et al. [33] found that those with recurrent PTSD were significantly more likely to have experienced academic burnout than those in the recovery trajectory, although there was no difference between the recurrent and delayed trajectories.

### Experiences during and immediately after the traumatic experience

The review also found mixed evidence for an association between peri-traumatic experiences and PTSD recurrence. The most consistent finding related to how stressful the traumatic experience was perceived to be at the time. For example, risk of recurrence was significantly higher in those with combat stress reactions [59] and in those with higher stress relating to the disaster they had experienced [56], as well as with greater trauma severity [49, 50]. However, recurrence was not found to be associated with subjective fear during the event [33]; directly witnessing a disaster [42]; property loss during the event [33, 42]; property damage [42]; displacement due to property damage [58]; near-death experience [58]; or having a family member injured, killed or missing [42, 58].

There was some evidence that initial post-traumatic stress symptoms immediately after the traumatic event could predict PTSD trajectory. Liang et al. [49, 50], in a study of PTSD in children from two schools affected by an earthquake, found that children from one of the two schools (‘School 2’) were significantly more likely to experience PTSD recurrence than children from the other school (‘School 1’). Further investigations revealed that after adjusting for immediate post-traumatic stress symptoms the school no longer predicted relapse; those from School 2 had significantly greater post-traumatic stress symptoms immediately after the disaster, which the authors suggest might be due to School 1 providing sufficient psychological services as well as having the same students and teachers before and after the earthquake (therefore perhaps greater social support available), whereas School 2 had insufficient psychological services and consisted of teachers and students from several different schools which could not be reconstructed after the earthquake.

One study [58] considered occupational-related covariates of PTSD recurrence for disaster recovery workers. They found that having mainly disaster-related occupational duties and lack of rest due to occupational duties were not associated with recurrence, but perceived poor workplace communication did predict recurrence.

### Post-trauma experiences and symptoms

An et al. [33] found that, compared to the delayed PTSD trajectory, those who experienced recurrence were less likely to have experienced post-traumatic growth after the traumatic event; however, there were no differences in post-traumatic growth between the recurrent and recovery groups. Fan et al. [42] found that neither positive coping nor negative coping six months post-disaster were associated with PTSD recurrence. In a military study, Karstoft et al. [48] found that poor adjustment to civilian life (i.e. difficulties with community reintegration after deployment) was significantly higher for the recurrent (‘relieved-worsening PTSD’) group than all other groups. However, it is not clear whether poor adjustment was a cause or an effect of PTSD symptoms worsening after initial improvement.

Two studies explored specific cluster symptoms. Murphy and Smith [54] found PTSD recurrence was not predicted by the magnitude of re-experiencing, avoidance, or hyperarousal symptoms. Boe et al. [15] found that the number of intrusion and avoidance symptoms five-and-a-half months post-trauma did not predict recurrence, but the number of intrusion and avoidance symptoms both fourteen months and five years after the disaster did predict recurrence.

### Social support

Only three studies directly considered social support as a potential covariate. Armenta et al. [36] found no association between social support and PTSD recurrence, and Perconte et al. [57] found that family support did not predict recurrence. Fan et al. [42] found that level of social support six months after experiencing an earthquake was not associated with PTSD recurrence, but those in the ‘relapsing’ group reported significantly less social support 24 months after the earthquake than those in the ‘recovery’ group.

### PTSD treatment

Most of the studies investigating treatment for PTSD found that not receiving interventions, or discontinuing treatment, were associated with PTSD recurrence. For example, Osenbach et al. [55] found that those who received ‘usual care’ only were significantly more likely to experience recurrence than those who received interventions designed to reduce post-traumatic symptoms. Davidson et al. [40] found that those who received placebo treatment were significantly more likely to experience recurrence than those who received fluoxetine. Martenyi et al. [53] found that those who discontinued fluoxetine treatment were significantly more likely to experience recurrence, especially for those with combat-related PTSD. However, Perconte et al. [57] found that number of weeks enrolled in treatment and number of treatment sessions attended did not significantly affect risk of recurrence. In this study, though, being hospitalised at least once since the termination of treatment was used as a proxy measure of ‘recurrence’ and so the findings are arguably not truly representative of actual recurrent episodes of PTSD. Overall, our findings indicated some evidence that treatment helped to avoid recurrent episodes.

## Discussion

In this study, we systematically reviewed 35 studies to identify definitions and prevalence of recurrent PTSD and factors associated with recurrence. It is important to define and operationalise recurrence as the concept needs to be understood in order to make prevention efforts. The health-related, social and economic costs of PTSD can be substantial. PTSD negatively affects individuals’ emotional wellbeing and physical health [7], impedes social relationships [69], limits productivity at work and increases sickness absence [70]. The direct costs (e.g., medical care costs) and indirect costs (e.g., costs of unemployment or reduced productivity) of PTSD can create substantial economic burden [7, 71]. Determining the predictors of recurrence of PTSD (which can only be properly understood if ‘recurrence’ itself has a clear definition) is important for prevention efforts: identifying those most at risk for recurrent episodes would allow for the subsequent investigation of ways of mitigating or preventing the risk. However, we found little consensus as to how recurrence is defined, mixed evidence on the prevalence of recurrence and inconsistent findings relating to predictors of recurrence. This lack of clarity about what relapse or recurrence is, and is not, is a major barrier to understanding this important topic.

In a previous review exploring PTSD recurrence in veterans, Berge et al. [22] acknowledge that there is no generally accepted or used definition of recovery relating to psychological trauma. The definition of recurrence used in their review was *the return of symptoms following a period of complete recovery, representing the start of a new and separate episode*. However, it is not clear what length of time is covered by ‘a period of complete recovery’ nor what ‘complete recovery’ means. How many days, weeks, or months does an individual need to be free of symptoms of PTSD in order to be considered truly recovered? Is ‘symptom-free’ the only definition of recovery, or is ‘not meeting the criteria for PTSD’ enough? Our own review revealed that there is little consensus as to what recurrence means and the parameters for its definition. Even the terminology used varied across studies, with ‘relapse’, ‘recurrence’, ‘reactivation’ and numerous other terms often used to describe what essentially appeared to be the same concept. There was no consensus as to how long an individual needed to be free of symptoms in order to be considered recovered, nor for how long symptoms needed to recur in order to be considered a recurrent episode. Most studies simply defined recurrence as a change in symptoms between assessments, meaning that whether or not an individual was defined as having a recurrent episode or not very much depended on the scores they reported at arbitrary time-points. Even minor symptom fluctuations could cause someone to change from being identified as a ‘case’ to ‘recovered’ and vice versa. Because PTSD tended to be examined using prospective studies where symptoms were assessed at predetermined assessment points, it is possible that individuals may have onsets of PTSD after one assessment and then remit before the next. With no retrospective assessment between time-points, it is difficult to assess the true prevalence of recurrence. Andrews et al. [16] make a similar point in relation to delayed onset PTSD, suggesting the absence of information about symptoms outside of the predetermined time-points of studies means that estimates of delayed onset PTSD may be unreliable.

The second aim of the review was to examine the prevalence of PTSD recurrence in existing literature. Given the numerous different ways of assessing PTSD, defining initial recovery and defining recurrence, as well as the differing time-points at which PTSD was assessed across studies, we suggest that the current data on recurrence prevalence is not especially meaningful. We found very different prevalence rates reported within the literature, with data suggesting that anywhere between 0.2% and 57% of trauma-exposed populations might experience recurrent episodes of PTSD. Some of the higher percentages we found seem greater than we would expect, given that only a minority of trauma-exposed people are likely to develop PTSD in the first place – let alone suffer from it, recover from it, and experience a recurrent episode. We would expect that studies carried out over a longer period of time would find higher recurrence rates, simply because in these studies there is more time for recurrent episodes to occur. However, the highest prevalence rate (57%) was found in a study which took place over only 27 weeks [43]; the authors labelled these participants as ‘intermittent cases’ and it appears likely that symptom fluctuation, rather than true recovery and recurrence, occurred in this study – and potentially many others. Additionally, studies did not typically control for exposure to subsequent trauma, meaning that ‘recurrences’ of PTSD identified may actually be new episodes, rather than a relapse. Further research studies, especially research involving assessments over a number of years, are needed to establish the true prevalence of recurrent PTSD which also needs to be clearly defined with an agreed time period between remission and relapse.

It has been proposed that recurrence rates might increase with old age. Murray [72] suggests that PTSD can be ‘reactivated’ in older age because physical illnesses become more common, which can reactivate traumatic memories; increased dependence on others due to ageing can reactivate feelings of helplessness; and loss of structure and identity caused by retirement can similarly reactivate traumatic symptoms. Other factors relating to ageing such as decline of cognitive function, difficulty controlling ruminations, reminiscing, and late-life stressors such as serious illness, surgical procedures and death of spouses, siblings or close friends can either directly remind the person of their previous traumatic experience(s) or can induce similar feelings of vulnerability [73]. Three studies of adults in this review did not find age predicted recurrence [35, 36, 57]; however, the populations trended young overall, with each of the three studies reporting the mean age of participants was under 40. We suggest, then, that more studies of older adults with lifetime PTSD are needed to establish whether this group are at increased risk of recurrence.

The third aim of this review was to understand factors associated with PTSD recurrence. Although a number of potential covariates were considered, most were not investigated by more than a few studies, and findings were varied and inconsistent. Of the covariates investigated by multiple studies, none were found to have significant associations with recurrence across all studies. It was therefore not possible to quantify the extent to which potential risk factors contribute to the risk of recurrence. One reason for the inconsistent findings might be the relatively small numbers of participants with recurrent PTSD in many of the studies. We note also that most studies did not consider either subsequent trauma or treatment impact in their analysis of predictors of recurrence.

We did not find strong evidence of an association between PTSD recurrence and comorbid psychiatric conditions. Recurrence of other mental health disorders, such as anxiety, is reportedly associated with comorbid psychiatric conditions including major depression, alcohol and substance use disorders [74]. Additionally, comorbid disorders have been found to be associated with an ‘unfavourable long-term course’ of PTSD [18]. However, in a review of predictors of developing PTSD, Brewin et al. [75] found that while psychiatric history was associated with development of PTSD, it was not a strong risk factor – factors operating during or after the traumatic exposure had greater effects than the pre-trauma factors. Many studies in this review found no evidence of a relationship between PTSD recurrence and other mental health conditions; in those that did find a relationship, it was not always clear whether the other conditions pre-dated the recurrent PTSD episode or not. Overall, the most consistent evidence we found indicated that recurrence of PTSD was associated with greater stress and traumatic response at the time of the traumatic experience.

We did not find evidence to suggest that trauma type may affect recurrence. Many studies examined PTSD trajectories after a single traumatic event. Those that did include participants who had experienced various different types of trauma did not consider trauma type as a potential predictor of recurrence. Given the wide variations in methodology, it was not appropriate for us to compare recurrence rates for different trauma types within the review. Future research should include participants who have experienced different types of trauma and should consider trauma type as a potential predictor of PTSD trajectory.

Only one study assessed PTSD during the COVID-19 pandemic, with Solomon et al. [62] reporting that 16.7% of initially-recovered participants experienced recurrence during the pandemic. However, it is not clear how many of this cohort may also have experienced recurrence *before* the pandemic, and without being able to make that comparison, we cannot ascertain the extent to which recurrence was exacerbated by the pandemic. Additionally, the percentage (16.7%) is similar to recurrence rates in several other, non-COVID studies. Ideally, future studies will present data on PTSD recurrence rates for one cohort at regular intervals, including data collected during or after the COVID-19 pandemic, to ascertain whether the pandemic did affect recurrence rates.

In their review, Steinert et al. [18] identified older age, higher education, greater trauma severity, higher baseline symptoms, more physical/functional impairments, and poorer social support as predictors of ‘unfavourable’ long-term course of PTSD. These were identified as predictors due to being reported in at least two studies within their review. The current review did not find consistent evidence that age, education, trauma severity, baseline symptoms, impairments or social support predicted recurrence – although age was only considered in studies of young people. We found some evidence from treatment studies that fluoxetine reduced the risk of recurrence, as did participation in an intervention involving a combination of motivational interviewing, behavioural activation and pharmacotherapy. It is therefore difficult to make recommendations relevant to occupational health, as we had hoped to do. Managers of trauma-exposed employees who have developed PTSD may have questions around whether recovered individuals can go back to frontline work, or whether they risk experiencing a recurrence of PTSD. Our findings tentatively suggest that recurrence might be relatively rare (rates of recurrence ranged from 0.2 − 57% in full trauma-exposed samples, mean 13.1%; 4.9 − 49.6% in PTSD-only subgroups, mean 24.5%; and 5.8 − 50% for recovered subgroups, mean 25.4%) but clearer definitions and assessments of recurrence are needed to substantiate that claim. As we found no consistent evidence of predictors of recurrence, it was therefore not possible to identify which sub-groups of people might be more likely to have their PTSD recur. We did find evidence from two studies that recurrence was more prevalent in groups of PTSD patients treated with placebos compared to PTSD patients treated with fluoxetine, suggesting that medication appears at least somewhat effective in reducing the risk of recurrence. However, we found no studies looking at the impact of first-line treatments on relapse (i.e. trauma-focused cognitive behavioural therapy [76] or eye movement desensitisation and reprocessing [77, 78]) which is a major gap in the literature. Whilst more, high-quality studies are carried out, employers should ensure that workers get evidence-based treatments and have an occupational mental health assessment on completion of potentially traumatic work to provide an expert judgement, given that we cannot identify any clear risk factors from the literature.

The key limitation of the literature on PTSD recurrence is that it is not always easy to differentiate between recurrence and symptom fluctuation, and it is also difficult to know what ‘recovery’ truly means. It is not clear how many of the so-called ‘recovered’ participants within the reviewed studies may have been close to clinical thresholds for PTSD at the assessment points. Rather than moving from distinct ‘recovered’ to ‘recurrent episodes’, it may be that individuals only experienced small fluctuations in PTSD symptoms, moving them above and below the symptom thresholds. Indeed, the authors of several of the included studies remarked on the difficulties in identifying PTSD trajectories. In Boe et al.’s [15] study, clinical interviews were conducted by two clinical psychologists who were trained and supervised by an experienced clinician and trauma researcher and even these experienced individuals had difficulties identifying recurrence of PTSD, with one case being recategorised from ‘full-blown PTSD reactivation’ to ‘sub-syndromal reactivation’ after discussion between the researchers. Markowitz et al. [52] pointed out that, as they defined relapse as ‘loss of response (to treatment) status’, relapse might reflect barely crossing that threshold: indeed, more in-depth analysis of their six ‘relapsers’ showed that all but one still showed some, albeit more modest, treatment benefit relative to their baseline PTSD severity.

Sakuma et al. [58] discussed their finding of a ‘fluctuating’ trajectory (and lack of a delayed-onset trajectory), differing from the typical four trajectories widely accepted within the PTSD literature. They suggested the difference may be due to variations in the duration of study periods and characteristics of the study samples. The majority of studies which produce the typical four trajectories are conducted over short periods between a few months and two years [9], compared to the longer (54-month) period of Sakuma et al.’s [58] study: the trajectory commonly identified as ‘delayed onset’ could really be a fluctuating trajectory if examined over a longer period. Or, it could reflect a gradual accumulation of symptoms resulting in a delayed *presentation* of PTSD, rather than delayed onset.

The time-points of assessments could also affect reported prevalence rates. For example, Sungur & Kaya [64] pointed out that some of their ‘recurrent’ cases would have been considered ‘recovered’ if the study period had been shorter or if participants had not been reassessed at the particular time-points chosen. They also noted that symptoms across the entire participant population seemed to be higher at particular times during the study (namely, at the anniversary of the event and at the time of a disappointing result of a court hearing for compensation), suggesting that the nature and course of PTSD might be influenced by particular events which might trigger unwanted memories of the traumatic event. In the current review, most studies assessed participants for at least a year, but not all: five [38, 39, 43, 52, 53] followed participants for less than a year. Additionally, two studies [44, 47] reported assessing participants pre-treatment and four months post-treatment but it was not clear how long treatment lasted.

We suggest that PTSD recurrence may not have been adequately assessed in many of the included studies. For example, Chopra et al. [39] described how, in order to minimise respondent burden, assessors were expected to stop inquiring about PTSD symptoms if participants were unlikely to meet the criteria and if they answered no to particular questions on the assessment tool. This could mean that some individuals who did have recurrent episodes of PTSD were not identified as they did not complete the full measures. Additionally, we found that a number of studies had very vague definitions of recurrence, such as ‘increasing symptoms’, where it was unclear what exactly this meant. Others used hospitalisation as a proxy measure for recurrence, or simply asked participants whether they perceived their symptoms had been exacerbated and in one case used the investigator’s own judgements as a way of determining recurrence. It is therefore likely that some recurrent cases may have been missed while others who never truly ‘recovered’ at all may have been reported to have experienced recurrence. Overall, the vague and inconsistent ways of assessing recurrence mean it is currently impossible to ascertain true recurrence rates within existing literature.

It is also possible that recurrent trajectories of PTSD appear in studies which do not identify them as such. For example, in Andrews et al.’s [16] review, the authors note that some cases of ‘delayed-onset PTSD’ in veterans of relatively old age with long intervals to first onset may in fact have had episodes of PTSD soon after their traumatic experiences which were undisclosed or forgotten. In other words, some cases of supposedly ‘delayed-onset’ PTSD might actually be recurrent cases. Andrews et al. [16] also point out that many of the studies included in their review of delayed-onset PTSD did not assess whether respondents could have had onsets of PTSD and then remitted before the next assessment point – which could lead to both over- and under-estimates of delayed-onset rates of PTSD. Indeed, the studies included in our own review tended to focus only on the scores at the various time-points and did not explore participants’ perceptions of symptom fluctuations outside of the time-points set by the study.

### Limitations

There are a number of limitations of the literature reviewed. Many did not collect data on whether participants had undergone any intervention or not, and those that did tended not to include this as a potential confounding variable. The majority of studies did not assess whether participants experienced additional potentially traumatic experiences between PTSD assessments. Many did not define the parameters of ‘recovery’ and ‘recurrence’ and it is not clear whether recurrent episodes identified were truly recurrent episodes or merely symptom fluctuations. Many did not collect data on whether or not participants received any treatment for PTSD between data collection time-points, and many of those which did ask participants whether they had received any treatment did not distinguish between types of treatment. It is therefore unclear if, and how many, participants in many studies received any evidence-based PTSD treatment or not. Additionally, the majority of studies did not collect data on the time period of any treatment received. Some studies had extremely long gaps (e.g., decades) between assessments which could mean that recurrences were missed.

There are also limitations of the review process itself. Firstly, the screening, data extraction and quality appraisal were carried out by one author. Although decisions about exclusion or inclusion were discussed with the second author, it would have been preferable to have multiple screeners. We limited the review to English-language studies only, meaning that important studies published in other languages would have been missed. We included only studies which identified ‘recurrent episodes’ (or equivalent terminology e.g. relapse, reactivation); studies which identified no recurrent trajectory were not reviewed. It may be that these studies did not include a sufficient number of assessments to pick up on recurrent episodes, but it may also be that no participants in these studies experienced recurrence and therefore the true prevalence of recurrence may be lower than this review suggests.

## Conclusions and implications

**Fig. 2:**
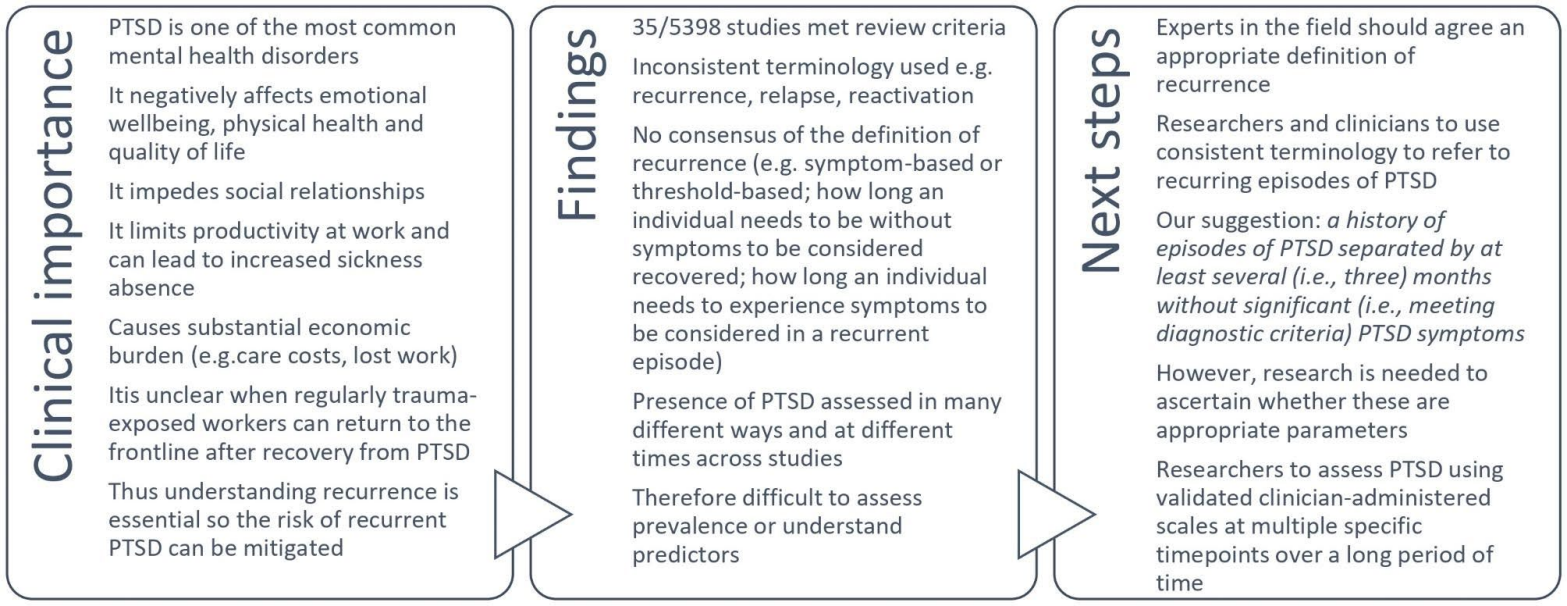
Summary of review and suggested next steps

The main conclusion that can be drawn from the current review is that, moving forward, better clarity and consensus regarding the definition and identification of recurrent PTSD are urgently needed. Berge et al. [22] suggest that consistent definitions of relapse-related terms, supported by empirical research, are required in order to make studies of PTSD trajectories more robust. The findings of this review support this suggestion. Experts in the field should agree on an appropriate definition of recurrence (i.e. symptom-based or threshold-based) and should agree how long an individual needs to be ‘better’ for in order to be considered recovered as well as how long an individual needs to experience symptoms for in order to be considered as having a recurrent episode. Recurrence is arguably better-defined for recurrent depressive disorder, with the ICD-11 stating that recurrence is characterised by a history of depressive episodes separated by *at least several months* without significant mood disturbance [79]. However, further clarity is still needed. How many months is ‘several’? What are ‘significant’ symptoms? Still, we suggest this might be a useful starting point for a working definition of recurrent PTSD: *a history of episodes of PTSD separated by at least several (i.e., three) months without significant (i.e., meeting diagnostic criteria) PTSD symptoms*. However, further research is necessary to clarify whether these parameters (i.e. three months as a time period, symptom thresholds as a diagnostic tool) are the most appropriate to use. Using consistent terminology within the literature would make it easier to researchers in the future to understand true prevalence rates of PTSD recurrence and to compare them across studies. Further research allowing for the identification of recurrent PTSD episodes is needed. We believe the gold standard for assessing PTSD and properly identifying its trajectories, including recurrent trajectories, would be using the Clinician Administered PTSD Scale (CAPS) [80], or other validated questionnaires, at multiple specific time points over a long period of time. Figure 2 summarises the findings of the review and the proposed next steps based on our findings.

It is important to understand recurrence in order to take steps towards reducing the risk of PTSD recurring. However, due to the inconsistent findings relating to predictors of recurrence, it is difficult to draw conclusions about the best ways of preventing or minimising recurrence. We suggest that ensuring that people who develop PTSD are provided with timely, evidence-based treatments is a logical first step [68]. Second, awareness of ‘early warning sign’ symptoms and ‘triggers’ might be useful, as well as awareness of effective coping strategies and how to access support. That is, if people with PTSD are able to recognise when they are struggling more and acknowledge that they need to be proactive in ensuring symptoms do not develop into full-blown PTSD again, they may be able to draw on their coping skills or reach out for formal or informal support when a recurrent episode seems imminent and may be able to stave off the recurrent episode. We also suggest that reframing the re-emergence of symptoms in a more positive way might be useful: instead of feeling defeated that symptoms have recurred, people could remind themselves that they have recovered once and therefore know that they are capable of doing so again. Within organisational settings, it is also important to foster an environment in which people who have any mental health condition, including PTSD, feel confident that asking for help will not lead to stigmatisation or increase the likelihood of inappropriate job loss. It may also be helpful to incorporate relapse prevention, understanding ‘warning signs’ of recurrent episodes and positive reframing into PTSD treatment programmes.

### Electronic supplementary material

Below is the link to the electronic supplementary material.

Supplementary Material 1

## Data Availability

All data generated or analysed during this study are included in this published article.

## Abbreviations

CAPS: Clinician-Administered PTSD Scale
MST: Military sexual trauma
MST: Military sexual trauma
NIH: National Institutes for Health
PRISMA: Preferred Reporting Items for Systematic Reviews and Meta-Analyses
PTSD: Post-Traumatic Stress Disorder

## References

[1] Benjet C, Bromet E, Karam EG, Kessler RC, McLaughlin KA, Ruscio AM (2016). The epidemiology of traumatic event exposure worldwide: results from the World Mental Health Survey Consortium. Psychol Med.

[2] Brooks SK, Greenberg N, Lax P (2022). Preventing and treating trauma-related mental health problems. Textbook of acute trauma care.

[3] Bonanno GA (2004). Loss, trauma, and human resilience: have we underestimated the human capacity to thrive after extremely aversive events?. Am Psychol.

[4] American Psychiatric Association. Diagnostic and statistical manual of mental disorders (fifth edition). ; 2013. 10.1176/appi.books.9780890425596.

[5] Messman-Moore TL, Cook NK. Posttraumatic stress disorder. In: H. S. Friedman, editor. Encyclopedia of mental health (second edition). San Diego: Academic Press; 2016. pp. 308 – 12.

[6] Spottswood M, Davydow DS, Huang H (2017). The prevalence of posttraumatic stress disorder in primary care: a systematic review. Harv Rev Psychiatry.

[7] Kapfhammer HP (2018). Acute and long-term mental and physical sequelae in the aftermath of traumatic exposure - some remarks on the body keeps the score. Psychiatr Danub.

[8] Peleg T, Shalev AY (2006). Longitudinal studies of PTSD: overview of findings and methods. CNS Spectr.

[9] Galatzer-Levy IR, Huang SH, Bonanno GA (2018). Trajectories of resilience and dysfunction following potential trauma: a review and statistical evaluation. Clin Psychol Rev.

[10] van de Schoot R, Sijbrandij M, Depaoli S, Winter SD, Olff M, van Loey NE (2018). Bayesian PTSD-trajectory analysis with informed priors based on a systematic literature search and expert elicitation. Multivar Behav Res.

[11] Santiago PN, Ursano RJ, Gray CL, Pynoos RS, Spiegel D, Lewis-Fernandez R (2013). A systematic review of PTSD prevalence and trajectories in DSM-5 defined trauma exposed populations: intentional and non-intentional traumatic events. PLoS ONE.

[12] Mota N, Bolton SL, Enns MW, Afifi TO, El-Gabalawy R, Sommer JL (2021). Course and predictors of posttraumatic stress disorder in the Canadian Armed forces: a nationally representative, 16-year follow-up study. Can J Psychiatry.

[13] Magruder KM, Goldberg J, Forsberg CW, Friedman MJ, Litz BT, Vaccarino V (2016). Long-term trajectories of PTSD in Vietnam-era veterans: the course and consequences of PTSD in twins. J Trauma Stress.

[14] Karamustafalioglu OK, Zohar J, Guveli M, Gal G, Bakirn B, Fostick L (2006). Natural course of posttraumatic stress disorder: a 20-month prospective study of Turkish Earthquake survivors. J Clin Psychiatry.

[15] Boe HJ, Holgersen KH, Holen A (2010). Reactivation of posttraumatic stress in male Disaster survivors: the role of residual symptoms. J Anxiety Disord.

[16] Andrews B, Brewin CR, Philpott R, Stewart L (2007). Delayed-onset posttraumatic stress disorder: a systematic review of the evidence. Am J Psychiatry.

[17] North CS, Oliver J (2013). Analysis of the longitudinal course of PTSD in 716 survivors of 10 Disasters. Soc Psychiatry Psychiatr Epidemiol.

[18] Steinert C, Hofmann M, Leichsenring F, Kruse J (2015). The course of PTSD in naturalistic long-term studies: high variability of outcomes. A systematic review. Nord J Psychiatry.

[19] Bonde JPE, Jensen JH, Smid GE, Flachs EM, Elklit A, Mors O, Videbech P (2022). Time course of symptoms in posttraumatic stress disorder with delayed expression: a systematic review. Acta Psychiatr Scand.

[20] Morina N, Wicherts JM, Lobbrecht J, Priebe S (2014). Remission from post-traumatic stress disorder in adults: a systematic review of long term outcome studies. Clin Psychol Rev.

[21] Pavlacic JM, Buchanan EM, McCaslin SE, Schulenberg SE, Young JN. A systematic review of posttraumatic stress and resilience trajectories: identifying predictors for future treatment of veterans and service members. Prof Psychol Res Pr. 2022;3266–75. 10.1037/pro0000451.

[22] Berge EE, Hagen R, Halvorsen JO (2020). PTSD relapse in veterans of Iraq and Afghanistan: a systematic review. Mil Psychol.

[23] Batelaan NM, Bosman RC, Muntingh A, Scholten WD, Huijbregts KM, van Balkom AJLM (2017). Risk of relapse after antidepressant discontinuation in anxiety disorders, obsessive-compulsive disorder, and post-traumatic stress disorder: systematic review and meta-analysis of relapse prevention trials. BMJ.

[24] Levy HC, O’Bryan EM, Tolin DF (2021). A meta-analysis of relapse rates in cognitive-behavioural therapy for anxiety disorders. J Anxiety Disord.

[25] Nagarajan R, Krishnamoorthy Y, Basavarachar V, Dakshinamoorthy R (2022). Prevalence of post-traumatic stress disorder among survivors of severe COVID-19 Infections: a systematic review and meta-analysis. J Affect Disord.

[26] Brooks SK, Webster RK, Smith LE, Woodland L, Wessely S, Greenberg N, Rubin GJ (2020). The psychological impact of quarantine and how to reduce it: Rapid review of the evidence. Lancet.

[27] Bonsaksen T, Heir T, Schou-Bredal I, Ekeberg Ø, Skogstad L, Grimholt TK (2020). Post-traumatic stress disorder and associated factors during the early stage of the COVID-19 pandemic in Norway. Int J Environ Res Public Health.

[28] Hori A, Sawano T, Ozaki A, Tsubokura M (2021). Exacerbation of subthreshold PTSD symptoms in a Great East Japan Earthquake survivor in the context of the COVID-19 pandemic. Case Rep Psychiatry.

[29] Moher D, Liberati A, Tetzlaff J, Altman DG (2009). Preferred reporting items for systematic reviews and meta-analyses: the PRISMA statement. BMJ.

[30] Braun V, Clarke V (2006). Using thematic analysis in psychology. Qual Res Psychol.

[31] Bagias C, Sukumar N, Weldeselassie Y, Oyebode O, Saravanan P (2021). Cord blood adipocytokines and body composition in early childhood: a systematic review and meta-analysis. Int J Environ Res Public Health.

[32] Rosella L, Bowman C, Pach B, Morgan S, Fitzpatrick T, Goel V (2016). The development and validation of a meta-tool for quality appraisal of public health evidence: Meta Quality Appraisal Tool (MetaQAT). Public Health.

[33] An YY, Huang JL, Yeung ETF, Hou WK (2022). Academic burnout and posttraumatic growth predict trajectories of posttraumatic stress disorder symptoms of adolescents following Yancheng Tornado in China. Int J Stress Manage.

[34] Andersen SB, Karstoft KI, Bertelsen M, Madsen T (2014). Latent trajectories of trauma symptoms and resilience: the 3-year longitudinal prospective USPER study of Danish veterans deployed in Afghanistan. J Clin Psychiatry.

[35] Ansell EB, Pinto A, Edelen MO, Markowitz JC, Sanislow CA, Yen S (2011). The association of personality disorders with the prospective 7-year course of anxiety disorders. Psychol Med.

[36] Armenta RF, Walter KH, Geronimo-Hara TR, Porter B, Stander VA, LeardMann CA (2019). Longitudinal trajectories of comorbid PTSD and depression symptoms among US service members and veterans. BMC Psychiatry.

[37] Benítez CIP, Zlotnick C, Stout RI, Lou FJ, Dyck I, Weisberg R, Keller M. (2012). A 5-year longitudinal study of posttraumatic stress disorder in primary care patients. Psychopathol. 2012;45(5):286 – 93. 10.1159/000331595. 10.1159/000331595 22797509

[38] Berntsen D, Johannessen KB, Thomsen YD, Bertelsen M, Hoyle RH, Rubin DC (2012). Peace and War: trajectories of posttraumatic stress disorder symptoms before, during, and after military deployment in Afghanistan. Psychol Sci.

[39] Chopra MP, Zhang H, Kaiser AP, Moye JA, Llorente MD, Oslin DW, Spiro IA (2014). PTSD is a chronic, fluctuating disorder affecting the mental quality of life in older adults. Am J Geriatr Psychiatry.

[40] Davidson JRT, Connor KM, Hertzberg MA, Weisler RH, Wilson WH, Payne VM (2005). Maintenance therapy with fluoxetine in posttraumatic stress disorder: a placebo-controlled discontinuation study. J Clin Pharmacol.

[41] DenVelde WO, Hovens JE, Aarts PGH, FreyWouters E, Falger PRJ, VanDuijn H (1996). Prevalence and course of posttraumatic stress disorder in Dutch veterans of the civilian resistance during World War II: an overview. Psychol Rep.

[42] Fan F, Long K, Zhou Y, Zheng Y, Liu X (2015). Longitudinal trajectories of post-traumatic stress disorder symptoms among adolescents after the Wenchuan Earthquake in China. Psychol Med.

[43] Gonçalves V, Jayson G, Tarrier N (2011). A longitudinal investigation of posttraumatic stress disorder in patients with Ovarian cancer. J Psychosom Res.

[44] Gross GM, Smith N, Holliday R, Rozek DC, Hoff R, Harpaz-Rotem I (2022). Racial disparities in clinical outcomes of Veterans affairs residential PTSD treatment between Black and White veterans. Psychiatr Serv.

[45] Hansen MB, Birkeland MS, Nissen A, Blix I, Solberg O, Heir T (2017). Prevalence and course of symptom-defined PTSD in individuals directly or indirectly exposed to terror: a longitudinal study. Psychiatry.

[46] Hepp U, Moergeli H, Buchi S, Bruchhaus-Steinert H, Kraemer B, Sensky T (2008). Post-traumatic stress disorder in serious accidental injury: 3-year follow-up study. Br J Psychiatry.

[47] Holliday R, Smith NB, Holder N, Gross GM, Monteith LL, Maguen S (2020). Comparing the effectiveness of VA residential PTSD treatment for veterans who do and do not report a history of MST: a national investigation. J Psychiatr Res.

[48] Karstoft KI, Armour C, Andersen SB, Bertelsen M, Madsen T (2015). Community integration after deployment to Afghanistan: a longitudinal investigation of Danish soldiers. Soc Psychiatry Psychiatr Epidemiol.

[49] Liang YM, Cheng J, Zhou YY, Liu ZK (2019). Trajectories of posttraumatic stress disorders among children after the Wenchuan Earthquake: a four-year longitudinal study. Eur J Psychotraumatology.

[50] Liang YM, Zhou YY, Liu ZK (2021). Consistencies and differences in posttraumatic stress disorder and depression trajectories from the Wenchuan Earthquake among children over a 4 year period. J Affect Disord.

[51] Madsen T, Karstoft K-I, Bertelsen M, Andersen SB (2014). Postdeployment suicidal ideations and trajectories of posttraumatic stress disorder in Danish soldiers: a 3-year follow-up of the USPER study. J Clin Psychiatry.

[52] Markowitz JC, Choo T-H, Neria Y (2018). Do acute benefits of interpersonal psychotherapy for posttraumatic stress disorder endure?. Can J Psychiatry.

[53] Martenyi F, Brown EB, Zhang H, Koke SC, Prakash A. Fluoxetine *v* placebo in prevention of relapse in post-traumatic stress disorder. Br J Psychiatry. 2002;181(4):315 – 20. doi:10/1192/bjp.181.4.315. 10.1192/bjp.181.4.315 12356658

[54] Murphy D, Smith KV (2018). Treatment efficacy for veterans with posttraumatic stress disorder: latent class trajectories of treatment response and their predictors. J Trauma Stress.

[55] Osenbach JE, Lewis C, Rosenfeld B, Russo J, Ingraham LM, Peterson R (2014). Exploring the longitudinal trajectories of posttraumatic stress disorder in injured trauma survivors. Psychiatry.

[56] Osofsky HJ, Weems CF, Hansel TC, Speier AH, Osofsky JD, Graham R (2017). Identifying trajectories of change to improve understanding of integrated health care outcomes on PTSD symptoms post Disaster. Fam Syst Health.

[57] Perconte ST, Griger ML (1991). Comparison of successful, unsuccessful, and relapsed Vietnam veterans treated for posttraumatic stress disorder. J Nerv Ment Dis.

[58] Sakuma A, Ueda I, Shoji W, Tomita H, Matsuoka H, Matsumoto K (2020). Trajectories for post-traumatic stress disorder symptoms among local Disaster recovery workers following the Great East Japan Earthquake: Group-based trajectory modeling. J Affect Dis.

[59] Solomon Z, Mikulincer M (2006). Trajectories of PTSD: a 20-year longitudinal study. Am J Psychiatry.

[60] Solomon Z, Garb R, Bleich A, Grupper D (1987). Reactivation of combat-related posttraumatic stress disorder. Am J Psychiatry.

[61] Solomon Z, Bachem R, Levin Y, Crompton L, Ginzburg K (2018). Long-term trajectories of posttraumatic stress disorder: categorical versus continuous assessment. Psychiatry.

[62] Solomon Z, Mikulincer M, Ohry A, Ginzburg K (2021). Prior trauma, PTSD long-term trajectories, and risk for PTSD during the COVID-19 pandemic: a 29-year longitudinal study. J Psychiatr Res.

[63] Sørensen HJ, Andersen SB, Karstoft KI, Madsen T (2016). The influence of pre-deployment cognitive ability on post-traumatic stress disorder symptoms and trajectories: the Danish USPER follow-up study of Afghanistan veterans. J Affect Disord.

[64] Sungur M, Kaya B (2001). The onset and longitudinal course of a man-made post-traumatic morbidity: survivors of the Sivas Disaster. Int J Psychiatry Clin Pract.

[65] Zanarini MC, Horz S, Frankenburg FR, Weingeroff J, Reich DB, Fitzmaurice G (2011). The 10-year course of PTSD in borderline patients and axis II comparison subjects. Acta Psychiatr Scand.

[66] Zlotnick C, Warshaw M, Shea MT, Allsworth J, Pearlstein T, Keller MB (1999). Chronicity in posttraumatic stress disorder (PTSD) and predictors of course of comorbid PTSD in patients with anxiety disorders. J Trauma Stress.

[67] Lai BS, Lewis R, Livings MS, La Greca AM, Esnard AM (2017). Posttraumatic stress symptom trajectories among children after Disaster exposure: a review. J Trauma Stress.

[68] National Institute for Health and Care Excellence. Post-traumatic stress disorder. 2018. https://www.nice.org.uk/guidance/ng116 31211536

[69] Campbell SB, Renshaw KD (2018). Posttraumatic stress disorder and relationship functioning: a comprehensive review and organizational framework. Clin Psychol Rev.

[70] Stergiopoulos E, Cimo A, Cheng C, Bonato S, Dewa CS (2011). Interventions to improve work outcomes in work-related PTSD: a systematic review. BMC Public Health.

[71] Davis LL, Schein J, Cloutier M, Gagnon-Sanschagrin P, Maitland J, Urganus A, Guerin A, Lefebvre P, Houle CR (2022). The economic burden of posttraumatic stress disorder in the United States from a societal perspective. J Clin Psychiatry.

[72] Murray A (2005). Recurrence of post traumatic stress disorder. Nurs Older People.

[73] Floyd M, Rice J, Black SR (2002). Recurrence of posttraumatic stress disorder in late life: a cognitive aging perspective. J Clin Geropsychol.

[74] Bruce SE, Yonkers KA, Otto MW, Eisen JL, Weisberg RB, Pagano M (2005). Influence of psychiatric comorbidity on recovery and recurrence in generalized anxiety disorder, social phobia, and panic disorder: a 12-year prospective study. Am J Psychiatry.

[75] Brewin CR, Andrews B, Valentine JD (2000). Meta-analysis of risk factors for posttraumatic stress disorder in trauma-exposed adults. J Consult Clin Psychol.

[76] Cohen JA, Mannarino AP, Deblinger E (2010). Treating trauma and traumatic grief in children and adolescents.

[77] Shapiro F (1989). Efficacy of the eye movement desensitization procedure in the treatment of traumatic memories. J Trauma Stress.

[78] Shapiro F (1996). Eye movement desensitization and reprocessing (EMDR): evaluation of controlled PTSD research. J Behav Ther Exp Psychiatry.

[79] World Health Organization. 6A71 Recurrent depressive disorder. In International statistical classification of diseases and related health problems (11th ed.). ; 2019. https://icd.who.int/browse11/l-m/en#/http%3A%2F%2Fid.who.int%2Ficd%2Fentity%2F1194756772.

[80] Weathers FW, Blake DD, Schnurr PP, Kaloupek DG, Marx BP, Keane TM. The Clinician-Administered PTSD Scale for DSM-5 (CAPS-5). [Assessment]. 2013. Available from www.ptsd.va.gov. 10.1037/pas0000486 PMC5805662 28493729

