# Supplementary material for: Recurrence of post-traumatic stress disorder: systematic review of definitions, prevalence and predictors
Source: BMC Psychiatry. 2024 Jan 9;24:37. doi: 10.1186/s12888-023-05460-x (PMC10777598; doi:10.1186/s12888-023-05460-x)
Supplement: Supplementary file 1 — Supplementary Material 1 [file 12888_2023_5460_MOESM1_ESM.docx]

**Appendix 1**

*Search strategy*

1. PTSD

2. “posttraumatic stress disorder”

3. “post-traumatic stress disorder”

4. “post traumatic stress disorder”

5. 1 or 2 or 3 or 4

6. risk factor*

7. prevalence

8. predictor*

9. 6 or 7 or 8

10. relapse*

11. recurren*

12. recurring

13. trajector*

14. course*

15. reactivat*

16. 10 or 11 or 12 or 13 or 14 or 15

17. 5 and 9 and 16

18. limit to humans

19. limit to English language
